# A nanoemulsion/micelles mixed nanosystem for the oral administration of hydrophobically modified insulin

**DOI:** 10.1007/s13346-021-00920-x

**Published:** 2021-02-11

**Authors:** Irene Santalices, Carlos Vázquez-Vázquez, Manuel J. Santander-Ortega, Victoria Lozano, Francisca Araújo, Bruno Sarmento, Neha Shrestha, Veronique Préat, Miguel Chenlo, Clara V. Alvarez, Federico Benetti, Juan Cuñarro, Sulay Tovar, Dolores Torres, María José Alonso

**Affiliations:** 1 Center for Research in Molecular Medicine and Chronic Diseases (CIMUS), University of Santiago de Compostela, Campus Vida, 15782 Santiago de Compostela, Spain; 2 Department of Pharmaceutics and Pharmaceutical Technology, School of Pharmacy, University of Santiago de Compostela, Campus Vida, 15782 Santiago de Compostela, Spain; 3 Department of Physical Chemistry, Faculty of Chemistry, University of Santiago de Compostela, Campus Vida, 15782 Santiago de Compostela, Spain; 4 Cellular Neuroanatomy and Molecular Chemistry of Central Nervous System Group, School of Pharmacy, University of Castilla-La Mancha, 02071 Albacete, Spain; 5 Regional Centre of Biomedical Research (CRIB), University of Castilla-La Mancha, 02071 Albacete, Spain; 6 Instituto de Investigação E Inovação Em Saúde (i3S), Instituto Nacional de Engenharia Biomédica (INEB), Universidade Do Porto, Rua Alfredo Allen 208, 4200-135 Porto, Portugal; 7 Instituto de Investigacão E Formacão Avançada Em Ciências E Tecnologias da Saúde (CESPU), 4585-116 Gandra, Portugal; 8 Advanced Drug Delivery and Biomaterials, Université Catholique de Louvain, Louvain Drug Research Institute, 1200 Brussels, Belgium; 9 Neoplasia & Endocrine Differentiation Group, Center for Research in Molecular Medicine and Chronic Diseases (CIMUS), University of Santiago de Compostela, Campus Vida15782, Santiago de Compostela, Spain; 10 ECSIN-European Center for the Sustainable Impact of Nanotechnology, ECAMRICERT SRL, Padova, Italy

**Keywords:** Oral, Peptide, Insulin, Nanocarrier, Nanoemulsion, Micelles

## Abstract

The potential of nanoemulsions for the oral administration of peptides is still in its early stage. The aim of the present work was to rationally design, develop, and fully characterize a new nanoemulsion (NE) intended for the oral administration of hydrophobically modified insulin (HM-insulin). Specific components of the NE were selected based on their enhancing permeation properties as well as their ability to improve insulin association efficiency (Miglyol 812, sodium taurocholate), stability in the intestinal fluids, and mucodiffusion (PEGylated phospholipids and poloxamer 407). The results showed that the NE co-existed with a population of micelles, forming a mixed system that exhibited a 100% of HM-insulin association efficiency. The nanosystem showed good stability and miscibility in different bio-relevant media and displayed an acceptable mucodiffusive behavior in porcine mucus. In addition, it exhibited a high interaction with cell mono-cultures (Caco -2 and C2BBe1 human colon carcinoma Caco-2 clone cells) and co-cultures (C2BBe1 human colon carcinoma Caco-2 clone/HT29-MTX cells). The internalization in Caco-2 monolayers was also confirmed by confocal microscopy. Finally, the promising in vitro behavior of the nanosystem in terms of overcoming the biological barriers of the intestinal tract was translated into a moderate, although significant, hypoglycemic response (≈ 20–30%), following intestinal administration to both healthy and diabetic rat models. Overall, this information underlines the crucial steps to address when designing peptide-based nanoformulations to successfully overcome the intestinal barriers associated to the oral modality of administration.

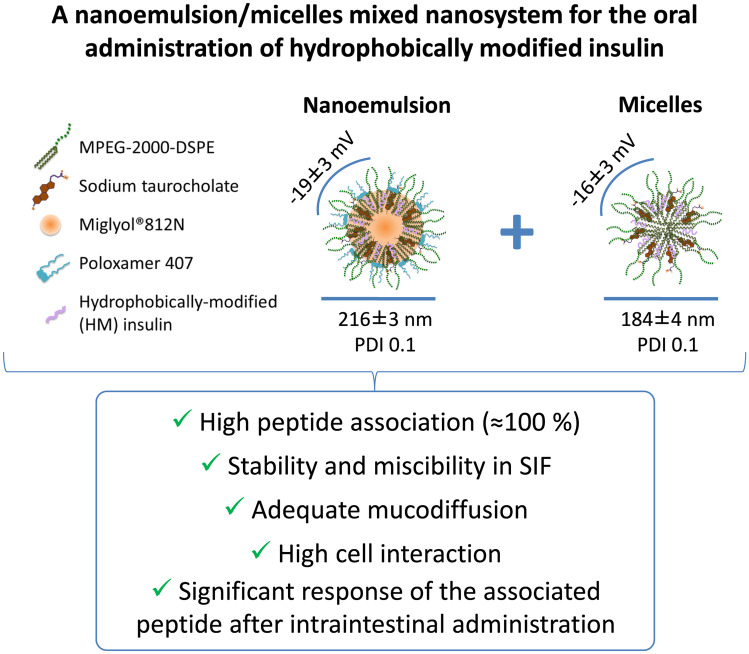

## Introduction

The increasing attention that therapeutic proteins and peptides are gaining in the pharmaceutical field is illustrated by the hundreds of them that have been approved by the FDA during the last decades [1, 2]. The attractiveness of these macromolecular drugs, which have opened up doors to many new and powerful therapies, relies on their specificity and potency. Despite of this, these macromolecules exhibit severe biopharmaceutical limitations, i.e., instability, short half-life, and limited oral bioavailability, which restrict their efficient clinical exploitation. One of their major constrains is their necessary administration by injection, a fact that is associated to a low patient compliance, especially in chronic treatments. Hence, the search for an alternative and non-invasive administration route for these molecules has become an attractive goal in the biopharmaceutical field [3, 4]. The growing global market for therapeutic peptides is investing considerable efforts in driving research to achieve the difficult goal of oral administration, which is usually the preferred choice, especially in the case of chronic treatments. In fact, in recent years, an important number of phase III clinical trials [5–7] have been initiated, which in addition to the already approved products, i.e., salmon calcitonin on 2012 [8], octreotide on 2015 [9], and semaglutide on 2019 [10, 11], will stimulate new oral peptide delivery strategies.

In principle, the oral route has been considered particularly desirable for the administration of insulin, since it would mimic the physiological pathway of the endogenously secreted insulin through the portal circulation [12, 13]. However, its high hydrosolubility and susceptibility to degradation by intestinal enzymes has made so far impossible its oral administration. Nanotechnology has been one of the strategies adopted for increasing the oral bioavailability of insulin [14–16]. Within this context, lipid-based nanocarriers have been outlined as particularly interesting based on their biocompatibility and also in the stabilizing and absorption enhancing properties of their lipid constituents [17]. In fact, the first nanocarrier that was shown to increase the oral absorption of insulin consisted of poly(alkylcyanoacrylate) (PACA) nanocapsules (NCs) [18]. Within this frame, our group has dedicated a great deal of effort to the design of a wide variety of lipid-based nanocarriers (i.e., nanoparticles [19] and nanocapsules [20–23]) that have shown a valuable potential for oral peptide delivery. Furthermore, we have recently showed the possibility of modulating the in vitro behavior of these systems by tuning their surface properties. Particularly, we have developed a nanoemulsion with improved stability, resistance to lipolysis and mucodiffusive properties [24]. An additional advancement in this area is the development of approaches to enhance the association of hydrophilic molecules to these lipid systems, such as the double emulsification, reverse micellization, hydrophobic ion pairing, or peptide-lipid/surfactant interactions. Despite these advances, the reported peptide loading capacity of these systems is usually lower than 1%, limiting their utility for oral administration [16, 17].

Considering this background information, the aim of this work was to rationally design, develop, and fully characterize a novel lipid-based nanosystem with the capacity to overcome the barriers associated to the oral administration of peptides. In this context, in order to increase the encapsulation of insulin and its permeability, we used a hydrophobically modified insulin (HM-insulin) (GRAVY ≈ − 0.0333). The resulting lipid nanosystem was extensively evaluated regarding its potential to deliver HM-insulin orally. Namely, the nanosystem was characterized in terms of (i) stability and miscibility in simulated intestinal fluids with and without enzymes, (ii) mucodiffusive properties, (iii) ability to interact with the intestinal cells, and (iv) potential to be used for the oral delivery of HM-insulin in diabetic rats.

## Materials and methods

### Materials

Hydrophobically modified insulin, named as HM-insulin (Mw 6256.23 Da), was kindly provided by Sanofi (Paris, France). Pharmaceutical grade poloxamer 407 (Kolliphor® P 407), sodium taurocholate (STC), and N-(carbonyl-methoxypolyethylene glycol-2000)-1,2-distearoyl-sn-glycero-3-phosphoethanolamine sodium salt (LIPOID PE 18:0/18:0-PEG 2000 (MPEG-2000-DSPE)) were purchased from BASF (Ludwigshafen, Germany), New Zealand Pharmaceuticals (Palmerston North, New Zealand), and Lipoid GmbH (Ludwigshafen, Germany), respectively. Caprylic/capric triglyceride (Miglyol® 812N) was purchased from Cremer, Oleo Division (Witten, Germany). The fluorescent dyes 1,10-dioctadecyl-3,3,30,30-tetramethylindodicarbocyanine perchlorate fluorescent dye (DiD oil) and 2-(6-hydroxy-3-oxo-(3H)-xanthen-9-yl)-5-isothiocyanatobenzoic acid (5-FITC,) were purchased from Life Technologies (Eugene, USA) and EMP GmbH Biotech (Berlin, Germany), respectively. Sodium glycocholate of pharmaceutical grade was purchased from New Zealand Pharmaceuticals (Palmerston North, New Zealand). Triton™ X-100 for molecular biology was obtained from Sigma-Aldrich (St. Louis, USA). Pancreatin (8× USP) was purchased from Biozym (Hamburg, Germany). Organic solvents were of HPLC grade, and all other products used were of high purity or reagent grade.

Human colorectal adenocarcinoma Caco-2 cells were purchased from American Type Culture Collection (Manassas, VA, USA) and used at passages 20–30. C2BBe1 human colon carcinoma Caco-2 clone, mucus secreting HT29-MTX and Human Burkitt’s lymphoma Raji B cell lines were purchased from the American Type Culture Collection (ATCC) and used at passages 53–64, 42–55, and X + 15–X + 20, respectively. The Hep G2 cell line was obtained from ECACC (UK, distributed by Sigma) and used at passages 9–12. Dulbecco’s modified Eagle medium (DMEM), fetal bovine serum (FBS), L-glutamine, non-essential amino acids (NEAA), penicillin and streptomycin, trypsin–EDTA, and Hank’s balanced salt solution (HBSS) were obtained from Gibco (Invitrogen Corporation, Life Technologies, UK). Eagle’s minimum essential medium (EMEM) was purchased from Sigma-Aldrich (St. Louis, USA), while phosphate-buffered saline (PBS) and Dulbecco’s phosphate-buffered saline with calcium and magnesium (DPBS) were purchased from Lonza (Basel, Switzerland). Reagents for cytotoxicity assays were ATPLite™ (Perkin Elmer, Massachusetts, MA, USA), Neutral Red based In Vitro Toxicology Assay Kit, LDH cytotoxicity detection kit plus (Roche, Mannheim, Germany), and MTT (3-(4,5-dimethylthiazol-2-yl)-2,5-diphenyltetrazolium bromide) reagent (Sigma-Aldrich, St. Louis, USA). For confocal studies, Transwell® polycarbonate inserts (6 wells, pore diameter of 3 μm, 4.67 cm^2^) were purchased from Corning (Madrid, Spain). For bioactivity cell studies, plasmids pMAXGFP, pSynSRE‐T‐luc, and pRSV‐βgal were respectively obtained from Lonza (Köln, Germany), Addgene (Cambridge, USA), and Promega (Madison, USA) while transfection reagents Viafect and Turbofect and were purchased from Promega (Madison, USA) and Thermo Fisher Scientific (USA), respectively, and the Passive Lysis Buffer 1X from Promega(Madison, USA).

### Preparation of HM-insulin-loaded mixed nanosystem

HM-insulin-loaded mixed system (nanoemulsion together with micelles) was prepared by the solvent displacement technique, as previously described [25]. Briefly, MPEG-2000-DSPE (15 mg) and Miglyol® 812 N (59 mg) were dissolved in 4.875 mL ethanol. Twenty-five microliters of an aqueous solution of sodium taurocholate (100 mg/mL) were then added to this lipidic phase followed by the addition of 1.5 mg HM-insulin dissolved in 100 μL 0.01 M HCl. This organic phase was vortexed and then immediately poured over 10 mL of an aqueous solution of poloxamer 407 (0.25% w/v) under magnetic stirring at 900 rpm, leading to the rapid formation of the nanoemulsion (NE) and micelles (HM-insulin and MPEG-2000-DSPE). Finally, ethanol was removed and the formulation was concentrated up to a final volume of 5 mL (Rotavapor Heidolph Hei-VAP Advantage, Schwabach, Germany). The blank nanosystem, used as control, was prepared by the same method without incorporating the HM-insulin.

For cell studies, fluorescent covalently linked FITC-HM-insulin was prepared. Briefly, HM-insulin was dissolved in 0.1 M sodium bicarbonate buffer (pH 9.5) at 5 mg/mL and 5-FITC in EtOH at 10 mg/mL. For each mL of HM-insulin solution, 50 μL of 5-FITC solution were dropwise added under magnetic stirring (900 rpm). After 1 h incubation at room temperature protected from light, the free 5-FITC was removed passing the mixture through 5 kDa Centri Pure P10 columns (Zetadex Gel Filtration columns—Centri Pure P10, EMP GmbH Biotech, Berlin, Germany). The pH of the conjugate FITC-HM-insulin solution was then adjusted to 6.8 (HM-insulin isoelectric point) with HCl 1 M to precipitate the conjugate. Then, the bicarbonate buffer was removed after centrifugation (5430 R Eppendorf Centrifuge, rotor F-35-6-30, Eppendorf AG, Germany) at 7197 g during 1 h at 4 °C, and the FITC-HM-insulin conjugate was dissolved in HCl 0.05 M to obtain a final HM-insulin concentration of 15 mg/mL. This solution was used to formulate the FITC-HM-insulin-loaded system as previously described. For mucodiffusion studies, fluorescently DiD-labeled mixed nanosystem was produced as above described with the addition of 80 μL of a 2.5 mg/mL ethanolic solution of DiD to the organic phase.

### Physicochemical, morphological, and drug loading properties of HM-insulin-loaded mixed nanosystem

The mean particle size and polydispersity index (PDI) of the nanocarriers were measured by dynamic light scattering using a Zetasizer® (NanoZS, ZEN 3600, Malvern Instruments, Worcestershire, UK) after diluting the samples with ultrapure water. Additionally, in order to get a more accurate size characterization, the light scattering ALV SP-86 goniometer (ALV 5000 Multi-tau correlator and a Coherent Sapphire optically pumped semiconductor laser operating at λ = 488 nm and 200 mW power) and the NanoSight NS3000 (laser operating at λ = 488 nm, Malvern Instruments, Worcestershire, UK) were also used to characterize the formulation through multi-angle dynamic light scattering (MA-DLS) and nanoparticle tracking analysis (NTA), respectively. MA-DLS measurements were performed at 25 °C (fixed temperature) and angles between 30 and 150° with increments of 10° [26]. NTA measurements were also performed at 25 °C by diluting the NE and the micelles in ultrapure water. This last technique was also used to quantify the concentration of NE and micelles in the mixed nanosystem. The ζ-potential was measured by laser-Doppler anemometry after diluting all the samples 33 times in 1 mM KCl (Zetasizer®).

The transmission electron microscopy (TEM CM 12, Philips, Netherlands) was used to analyze the shape of the separate species that define the mixed system (NE and micelles). For TEM analysis, samples were diluted with ultrapure water 1:50 and deposited on copper grids and stained with 2% (w/v) phosphotungstic acid solution, allowed to dry and then viewed under the TEM.

Atomic force microscopy (AFM) was used to analyze the topography of the mixed nanosystem and its separate species. For AFM analysis, samples were diluted with ultrapure water 1:1000 or 1:5000, deposited onto a mica substrate (SPI Supplies, Grade V-1 Muscovite), allowed to dry and then, viewed using a XE-100 instrument (Park Systems, Korea) with a non-contact silicon cantilever probe with high resonant frequency (325 kHz) and backside aluminum reflex coating (ACTA, supplied by Park Systems) [27].

The percentage of HM-insulin associated to the nanosystem was directly determined after extracting the HM-insulin from the mixed nanosystem by their complete disruption. For digestion, the HM-insulin-loaded mixed nanosystem was vortexed together with a combination of acetonitrile, Triton™ X-100 and 0.05% formic acid using a 2:1:1:16 proportion until obtaining a clear solution. To distinguish the amount of HM-insulin associated either to the NE or to the micelles, the same treatment was carried out after the separation of both species present in the mixed nanosystem by ultracentrifugation, process that was already reported to not affect the insulin bioactivity [21, 23]. For that purpose, 1 mL of the mixed nanosystem was ultracentrifuged at 84,035g for 1 h at 15 °C (Beckman Coulter, Optima L-90K, 70.1 Ti rotor, Brea, USA), obtaining a cream consisting of the isolated NE and an undernatant containing the micelles. Then, both fractions were collected separately and diluted with ultrapure water up to 1 mL to maintain the same concentrations as before, and then, they were disrupted using the treatment above explained. Ultra performance liquid chromatography (Acquity UPLC, Waters, Spain) with a C18 column as stationary phase (Acquity UPLC BEH C18 300 Å, 1.7 µm 2.1 × 50 mm, Waters, Spain) at 40 °C, coupled with a UV detector set at 215 nm, was used for the HM-insulin analysis. For this purpose, 5 μL of each sample at 10 °C were injected in duplicate and the flow rate was set to 0.5 mL/min. The gradient was obtained by mixing two mobile phases: 0.05% formic acid in ultrapure water (phase A) and 0.035% formic acid in acetonitrile (phase B) (Online resource 1).

The AE (%) of HM-insulin was calculated according to the following equation (Eq. 1):

1 $$AE\left(\mathrm{\%}\right)=\frac{\text{HM-insulin} \ \text{in the disrupted nanocarrier}}{\text{Total HM-insulin}} \times 100$$

where *HM-insulin in the disrupted* nanocarrier is the HM-insulin concentration determined by UPLC after treating the nanosystem for its disruption, and *Total HM-insulin* is the theoretical total HM-insulin concentration in the formulation. Analysis was done at least in triplicate.

The LC (%) was calculated as follows (Eq. 2):

2 $$LC \left(\mathrm{\%}\right)= \frac{\text{Total HM-insulin}\times \mathrm{ AE }}{\text{Total weight of nanocarrier}} \times 100$$

where *Total HM-insulin* is the theoretical total HM-insulin concentration in the formulation, *AE is* the HM-insulin association efficiency and *Total weight of nanocarrier* is the weight of the nanocarrier calculated using its yield. For calculating the total weight of both the mixed nanosystem and its separate species, they were separately lyophilized. Then, by the difference of the weight before and after the freeze-drying process, the yield of the process was determined. Analysis was done in triplicate.

### Stability of HM-insulin formulated in the mixed nanosystem during storage

The colloidal stability of the HM-insulin-loaded mixed nanosystem was evaluated under three different storage conditions: 4 °C, room temperature ≈ 25 °C, and 40 °C, following recommendations stated in the ICH (International Council for Harmonization of Technical Requirements for Pharmaceuticals for Human Use) Stability Guidelines. For that purpose, the particle size, PDI and ζ-potential of the formulation were monitored up to 6 months (0.5, 1, 2, 3, and 6 months) to check its potential colloidal destabilization. The amount of HM-insulin that remained associated to the nanosystem after 3 months storage was also determined. Analysis was done in triplicate.

### Interaction of HM-insulin formulated in the mixed nanosystem with bio-relevant media: miscibility, colloidal stability, and HM-insulin release

The colloidal stability of the HM-insulin-loaded nanosystem was evaluated by monitoring its particle size by DLS (Zetasizer®) during 4 h incubation under moderate horizontal shaking (300 rpm, Heidolph Instruments GmbH&Co. KG, Schwabach, Germany) at 37 °C with different gastrointestinal media (dilution 33.33) (composition detailed in Online resource 2) [28, 29]. To evaluate the effect of the intestinal enzymes, SIF supplemented with 1% pancreatin (8 × USP) was also prepared and centrifuged at 5000g for 5 min at 5 °C (5430 R Eppendorf Centrifuge, rotor F-35-6-30) to remove pancreatin aggregates before its incubation with the nanosystem [21, 30]. PDI and derived count rate were also monitored (data not shown). This study was done at least in three different batches in triplicate.

The amount of HM-insulin released from the mixed nanosystem upon their contact with SIF was also evaluated. For this purpose, the mixed nanosystem was ultracentrifuged at 84,035g for 1 h at 15 °C (Beckman Coulter, Optima L-90K, 70.1 Ti rotor). The same procedure was done with free HM-insulin as a control. The HM-insulin present in the fractions obtained (cream, undernatant, and precipitate) was determined by UPLC as follows. Free HM-insulin present in the undernatant was directly quantified while HM-insulin associated to the system in the different fractions was quantified after disrupting NE and micelles by treating them as previously explained*.* The precipitates obtained were solubilized in 500 µL of 0.05% (v/v) formic acid to be injected in the UPLC. These studies were done in triplicate.

Additionally, the miscibility of the system with SIF was also evaluated to predict if once in the intestine, the system is going to be able to mix well with the intestinal fluids being homogenously distributed along the gastrointestinal tract. For this purpose, and mimicking the in vivo dilution in rats when orally administered [31], 300 µL of the nanosystem were slowly added over 3.2 mL of SIF (water content in the gastrointestinal tract of fasted rats) and the appearance of the mix was monitored.

### Assessment of the mucodiffusive behavior of the HM-insulin formulated in the mixed nanosystem and in its separate species

The mucodiffusive behavior of the mixed nanosystem as well as of both separate species was assessed by multiple particle tracking analysis (MTA) using fresh porcine intestinal mucus, obtained from the local slaughterhouse, as a model of the human intestinal mucosa. For that purpose, the mixed nanosystem was labeled with DiD as previously described. Briefly, 5 µL of the DiD-loaded nanosystem previously diluted in water (1/20) were mixed with 100 µL of mucus. Sample volumes of 10 μL were placed in a chamber created by the placement of a 120-μm-thick, double-sided adhesive sticker between a microscope slide and a cover glass. For each sample, more than 20 videos of 800 frames were recorded at a frame rate of 100 fps (> 100 trajectories per video) using an Andor Zyla 4.2 camera and a PLAN APO 100X 1.4 oil-immersion objective always focused at 12–16 µm above the cover glass (Nikon microscope, Izasa Scientific, Spain). The diffusion coefficient of each particle was calculated offline according to the following equation: (Eq. 3): ${\langle MSD \rangle}=4{D}_{e}{\tau }^{\alpha }$ by fitting the mean square displacement 〈MSD⟩ as a function of the time. Being D_e_ the effective diffusion coefficient, α the anomalous exponent that gives information about the nature of the diffusion mode of the nanosystem in mucus (active transport (α ≈ 2); superdiffusion (0.9 ≤ α < 2); subdiffusion (0.4 ≤ α < 0.9); hindered diffusion (0.2 ≤ α < 0.4); immobilization (0.2 < α)) and τ, the time scale, which is the time during which particles were allowed to move before calculating their displacement trajectories (fixed at 1 s for these experiments (100 frames/s)) [24, 32–35].

Both polystyrene nanoparticles (mucoadhesive) and poloxamer 407-coated polystyrene nanoparticles (mucodiffusive) were used as controls. Additionally, in order to ensure that the signal observed did not originate due to the released DiD, the same procedure was performed using an aqueous solution of DiD at the same concentration as in the nanosystem.

### In vitro cell culture studies of HM-insulin formulated in the mixed nanosystem

#### Colloidal stability and HM-insulin release in cell culture media

Since cytotoxicity and bioactivity cell studies were performed using supplemented-DMEM and supplemented-EMEM, the colloidal stability of the mixed nanosystem during 24 h of contact with these cell media was evaluated. In the same line, taking into account that confocal and flow cytometry (FACS) were performed in HBSS, the colloidal stability of the mixed nanosystem was also evaluated in this medium . Additionally, HM-insulin released from the mixed nanosystem upon 20 h of contact with supplemented-EMEM and upon 4 h of contact with HBSS was evaluated following the procedure described above. These studies were done in triplicate.

#### Cell Cultures

Caco-2 cells, C2BBe1 human colon carcinoma Caco-2 clone cells, mucus-producing HT29-MTX cells and Human Burkitt’s lymphoma Raji B cells were separately cultured in flasks containing DMEM high glucose w L-Glutamine cell culture medium (CCM) supplemented with 10% FBS, 1% NEAA, and 1% penicillin (100 U/mL)/streptomycin (100 μg/mL) (v/v). Cells were maintained at 37 °C in a humidified incubator (Binder, Germany) under a controlled atmosphere with 95% of relative humidity (HR) and 5% CO_2_. CCM was renewed every 2–3 days, and cells were passaged when 70–80% confluence was achieved by trypsinization. HepG2 cells were grown in EMEM supplemented with 10% FBS, 1% NEAA, 2 mM L-glutamine, 1% penicillin (100 U/mL)/streptomycin (100 μg/mL) at 37 °C, 95% of HR and 5% CO_2_ and passaged by trypsinization weekly.

#### Cytotoxicity studies of both blank and HM-insulin-loaded nanosystem, in Caco-2, C2BBe1, and HT29-MTX cells (ATP, NRU, LDH, and MTT)

For each technique at least *n* = 3 independent experiments with *n* = 3 formulation and *n* = 3 replicates per condition were performed. For all experiments, different concentrations of both blank and HM-insulin loaded nanosystem were tested. Water and CCM were respectively used as controls of the nanosystem vehicle and untreated cells. Plates were incubated on the dark at 37 °C, 90–95% HR and 5% CO_2_.

**Cell culture seeding.** Cell cultures were maintained in supplemented-DMEM and seeded (200 µL/well) at a density of 1 × 10^4^ cells/well into flat-bottom 96-well plate for ATP, NRU, and LDH assays and 2 × 10^4^ cells/well in the case of MTT studies. Plates were incubated for 24 h to allow cell attachment prior the cytotoxicity studies. All assays were done at least in triplicate, and EC50 values were calculated using the GraphPad Prism 7 program (California, USA).

**Adenosine triphosphate (ATP) cell viability assay in Caco-2 cells.** ATPLite™ (luminescence-based method) was used to measure intracellular ATP, which decreases rapidly when cells undergo necrosis or apoptosis. Twenty-four hours after cell seeding, CCM was replaced with 200 µL of (i) nanosystems, (ii) controls, and (iii) 1 µM staurosporine solution from Streptomyces (positive control). Then, after 2 and 24 h incubation, cells were washed with PBS. Straightaway, 100 µL of new PBS and 50 μL of mammalian cell lysis solution were added per well. After 5 min, 150 μL of cell lysates were transferred into a white flat-bottom 96-well plate. For the standards, 10 μL of each, 90 μL of PBS, and 50 μL mammalian cell lysis solution were added per well. For blank wells, 100 µL of PBS and 50 μL of mammalian cell lysis solution were added per well. Finally, 50 μL of substrate solution were added to all wells and plates were shaken for 5 min. Measurements were performed at 22 °C in a luminometer Synergy 4 microplate reader (BioTek Instruments, Inc., Winooski, USA).

**Neutral red uptake (NRU) in Caco-2 cells.** This assay measures living cells via the uptake of the vital dye neutral red (Basic Red 5, Toluylene Red) and its incorporation into lysosomes. twenty-four hours after cell seeding, CCM was replaced with 200 µL of (i) nanosystems, (ii) controls, and (iii) 100 µg/mL of SDS (positive control). Then, after 2 and 24 h incubation, cells were washed with DPBS and 100 µL of a solution of 90% CCM and 10% Neutral Red (0.33% solution in DPBS) were added per well. After 3 h, cells were washed twice with DPBS and 100 µL/well of Neutral Red Assay Solubilization Solution were added. Plates were then shaken during 45 min, and absorbance was measured at λ = 540 nm using a Synergy 4 microplate reader (BioTek Instruments, Inc). The background absorbance at λ = 690 nm was subtracted from the measurements [21, 30].

**Lactate dehydrogenase (LDH) in Caco-2 cells.** Plasma membrane damage was quantified by measuring the release of cytoplasmic LDH (colorimetric cytotoxicity detection kit plus LDH). Twenty-four hours after cell seeding, CCM was replaced with 200 µL of (i) nanosystems, (ii) controls, and (iii) CCM to LDH (positive control). Then, 15 min before the end of the incubation (2 and 24 h), 10 µL of LDH lysis solution were added into the positive control wells. After incubation, plates were centrifuged 5 min at 300*g* (5810 R Eppendorf centrifuge, Eppendorf swing-bucket A-4-81 rotor with HL026 adapter, Eppendorf AG, Germany). Then, 50 µL of supernatant from all wells were transferred into another 96-well plate and 50 µL of working reagent were added to all wells. After 20 min, 25 µL/well of LDH stop solution were added and plates were shaken 30 s before reading the absorbance at λ = 500 nm with reference at λ = 750 nm using a Synergy 4 microplate reader (BioTek Instruments, Inc.) [21, 30].

**MTT cell viability assay in C2BBe1 and HT29-MTX cells.** MTT tetrazolium reduction assay was performed to determine the direct cytotoxicity of the nanosystem by decreasing the cell metabolic capacity. Twenty-four hours after cell seeding, CCM was replaced with 200 µL of (i) nanosystems, (ii) controls, and (iii) 1% Triton™ X-100 (positive control) wells. After 3 and 24 h of incubation, cells were washed with 200 µL of PBS and 200 µL/well of MTT solution (0.5 mg/mL) were added. After 4 h incubation, the excess solution was removed and formazan was solubilized with 200 µL/well DMSO during 20 min under horizontal shaking. Finally, the absorbance was measured at λ = 590 nm using a Synergy™ Mx Monochromator-Based Multi-Mode Microplate Reader (BioTek Instruments, Inc.) taking as reference the absorbance measured at a wavelength of 630 nm [36].

#### Interaction of FITC-HM-insulin-loaded mixed nanosystem with Caco-2 and C2BBe1 cell mono-cultures and with C2BBe1/HT29-MTX co-culture

After confirming the colloidal stability of the nanosystem and its negligible HM-insulin release in HBSS after 4 h at 37 °C, the interaction of the FITC-HM-insulin-loaded nanosystem with the intestinal cells was studied using FACS (BD FACSVerse™ flow cytometer, Becton Dickinson Biosciences, San Jose, CA, US) and confocal microscopy (Cell Observer Spinning Disk, Zeiss, Germany).

FACS analysis was performed in Caco-2 and C2BBe1 human colon carcinoma Caco-2 cell monocultures, as well as in C2BBe1 human colon carcinoma Caco-2 clone/HT29-MTX (9:1) co-culture. For this purpose, cells were seeded at a density of 3 × 10^5^ cells/well (6-well plates). After 48 h of incubation, cells were washed twice with HBSS, and 0.5 mL of the fluorescently labeled nanosystem at 0.3 mg/mL were added to each well. Control wells were prepared by adding either 45 µg/mL of free FITC-HM-insulin or HBSS. After 3 h incubation at 37 °C, cells were washed with HBSS, trypsinized and resuspended in 500 µL of HBSS. The fluorescence corresponding to the nanosystem and free FITC-HM-insulin was measured at λ = 519 nm. For each sample, 10,000 events were collected and FlowJo (TreeStar, USA) software was used for analyzing the samples.

For confocal microscopy, Caco-2 monolayers were cultured by seeding 5 × 10^5^ cells/well in Transwell® inserts and replacing the CCM on alternate days until 21 days. Then, monocultures were washed twice with HBSS and the integrity of the cell monolayers was confirmed by measuring the transepithelial electrical resistance values (TEER) using an EVOM epithelial voltammeter equipped with “chopstick” electrodes (World Precision Instruments, Sarasota, FL, USA). Four hundred microliters of 0.3 mg/mL FITC-HM-insulin-loaded mixed nanosystem were added to the apical side of the Transwell®, and HBSS was added to the basolateral chamber. Control wells were prepared with either 45 µg/mL of free FITC-HM-insulin or HBSS. After 2 h of incubation at 37 °C, cell inserts were washed twice with HBSS, fixed with 4% paraformaldehyde, and stored at 4 °C. Afterwards, inserts were gently washed in PBS and actin filaments were stained by adding 200 μL of rhodamine–phalloidin solution to the apical chamber (20 min, dark, room temperature) to reveal cell borders. Subsequently, the Transwell® membranes were mounted on glass slides, cell nuclei were stained with DAPi in Mowiol (1:5000) and coverslips were placed over the monolayers avoiding any bubbles. Mowiol® was then allowed to polymerize for 24 h at room temperature in dark [37]. Finally, monolayers were visualized under a Zeiss™ confocal microscope (LSM 150). Data were analyzed by the Axio Vision software (version 4.8) to obtain y–z, x–z, x–y, and z-stack views of the cell monolayers.

#### Bioactivity of HM-insulin formulated in the mixed nanosystem

After confirming the colloidal stability of the nanosystem and the amount of HM-insulin released upon 20 h of contact with and supplemented-EMEM at 37 °C, the preservation of the bioactivity of HM-insulin after its association to the nanosystem was evaluated using an assay based on promoter activation of an insulin early target gene. For this purpose, Hep G2 cells (enriched in insulin receptor expression [38]) were transfected with two different DNA plasmids*: *(i) pSynSRE‐T‐luc as promoter plasmid, which contains the − 324 to − 225 bp fragment of the hamster HMG-CoA synthase promoter with the SRE elements upstream of the minimal HMG-CoA synthase TATA box (− 28 to + 39) [39, 40]. This plasmid was chosen because it has been already proved that insulin regulates HMG-CoA synthase expression through those SRE sites in human cells [41]; and (ii) pRSV‐βgal as transfection control. After comparing the transfection efficiency of the commercial plasmid pMAXGFP with Viafect (> 95%) and Turbofect (≈ 65%), Viafect was selected as transfection reagent for this experiment.

MW48 multiwell plates were coated with 40 µL/well type I collagen solution (100 µg/mL) in PBS and washed three times with PBS. Hep G2 were trypsinized, resuspended in growth medium, seeded at a density of 2.5 × 10^4^ cells/well and allowed to grow for one full day. On the day of the transfection, a mixture containing 35 ng/well of pSynSRE‐T‐luc, 50 ng/well of pRSV‐βgal, 1.5 μL/well of Viafect and non-supplemented-EMEM up to a final volume of 25 μL/well was added to the plates. After 6 h of incubation, plates were washed three times with PBS and replaced by 400 μL/well of culture deprivation medium (growth medium with only 0.5% FBS and supplemented with 2 mM metformin) to help to reduce the basal luciferase expression while the cells were under transfection. After 4 h incubation, 100 μL/well of either different concentrations of HM-insulin (10, 100, 200, and 400 µIU/mL) or corresponding controls (HM-insulin solution, HM-insulin-loaded mixed nanosystem, blank mixed nanosystem and blank nanosystem later spiked with HM-insulin) were added and plates were incubated for 20 h. Each condition was tested in 6–8 replicates. Thereafter, plates were washed three times with PBS and 40 μL/well of Passive Lysis Buffer 1X were added. Finally, in order to perform the two enzymatic analyses, the lysate was divided as follows [42]: (i) 20 μL/well were transferred to a 96-well flat‐bottom white plate for luciferase assay and 40 μL/well of Luciferase Assay Buffer (Online resource 3) were added. Meanwhile, a positive control (mix of previous successful transfections) and a blank (buffer) were included in the plate. A luciferin solution (Online resource 3) was prepared as substrate and set at the injector. The luminescence reader program was set to add 35 µL/well of luciferin solution and read 5 s/well using a luminometer Mithras microplate reader (LB940, Berthold, Bad Wildbad, Germany)*; *(ii) 20 μL/well were transferred to a 96-well plasticware for the β‐galactosidase assay and 40 μL of Buffer ONPG (Online resource 3) together with 180 μL Buffer Z (Online resource 3) were added per well. Plates were incubated at 37 °C until the mixture turned yellow (around 10 min) and then, 75 μL/well of Buffer Stop (Na_2_CO_3_ 1 M) were added. Plates were read in the spectrophotometric setup at 490 nm in a Mithras microplate reader (LB940, Berthold, Bad Wildbad, Germany).

Five independent experiments were carried out. To obtain final data, results were normalized respect to the negative control (blank mixed nanosystem), being expressed as increments (∆Luc/β-gal) respect to this value.

### In vivo hypoglycemic response of HM-insulin formulated in the mixed nanosystem

All animal experiments were reviewed and approved by the ethics committee of the University of Santiago de Compostela ID: 1500AE/12/FUN01/FIS02/CDG3 (Spanish Royal Decree 1201/2005, of October 10th) and ID: 15010/17/17002 (Spanish Royal Decree 53/2013, of February 1st) and were executed in accordance with governing Spanish law and European Directives and Guidelines for the use of animals in animal studies; performed therefore in compliance with the Directive 2010/63/EU of the European Parliament and Council of 22nd September 2010 on the protection of animals used for scientific purposes and under the Spanish Royal Decree 53/2013 February 1st on the protection of animals used for experimental and other scientific purposes, including teaching.

Male healthy Sprague–Dawley rats were obtained from the Central Animals House of the University of Santiago de Compostela (Spain), kept under 12 h light/12 h dark cycles and fed a standard laboratory rodent diet (Panlab A04, Panlab laboratories). Blood samples were withdrawn from the tail vein 30 min prior to the experiments and measured using a hand-held glucometer (Glucocard™ G+ meter, Arkray Factory, Japan) to establish the baseline blood glucose levels. Initial acceptable glucose levels were established at ≥ 70 mg/dL for healthy animals and ≥ 250 mg/dL in the case of diabetic rats. During experiment, blood samples were collected at the following time points: 0.5, 1, 1.5, 2, 3, 4, 5, 6, 7, and 8 h after administration, in order to monitor the blood glucose levels following the different treatments.

#### HM-insulin dose–response and bioactivity after its inclusion in the mixed nanosystem following subcutaneous (SC) administration to healthy rats

Rats (average weight 313 ± 27 g) were fasted for 4 h prior to the experiments, which were carried out without anesthesia, with free access to water. Two different doses of HM-insulin-loaded mixed nanosystem and HM-insulin solution, 1 and 2 IU/kg (*n* = 8 and *n* = 13, respectively), were administered subcutaneously (SC) in a ratio of 1 μL/1 g rat. The area above the curves (AAC) representing the hypoglycemic were calculated (GraphPad Prism 7 program) to estimate the overall response obtained.

#### Effect on blood glucose levels following intrajejunal (IJ) administration of 100 IU/kg of HM-insulin-loaded mixed nanosystem to healthy rats

Rats (average weight 298 ± 19 g) were fasted for 4 h prior to the experiments, which were carried out without anesthesia, with free access to water. HM-insulin-loaded mixed nanosystem was intrajejunally (IJ) administered to the rats through a cannula that was surgically implanted into their jejunum. In the surgery, the proximal ends of the catheters were subcutaneously addressed to the back of the neck, and rats were daily weighed and monitored during 1 week to ensure their complete recovery. The HM-insulin-loaded mixed nanosystem was administered in a maximum volume of 0.3 mL at an HM-insulin dose of 100 IU/kg body weight (*n *= 8). The same dose of blank nanosystem was administered as control following the same procedure (*n* = 6). Additionally, 2 IU/kg body weight of an HM-insulin solution in saline were subcutaneously administered to a different control group (*n *= 6).

#### Effect on blood glucose levels following intraduodenal (ID) administration of 100 IU/kg of HM-insulin-loaded mixed nanosystem to diabetic rats

After 12 h fasting with free access to water, a single intraperitoneal streptozocin injection in 50 mM sodium citrate buffer at pH 4.5 was administered to healthy rats (average weight 223 ± 81 g) at a dose of 60 mg/kg body weight to render them diabetic. After this procedure, animals were kept under 12 h light/12 h dark cycles and fed on a standard laboratory rodent diet, while their blood glucose levels were daily monitored. Afterwards, 1 IU/kg body weight of HM-insulin was subcutaneously administered to rats with blood glucose > 500 mg/dL and a second intraperitoneal streptozocin injection was administered to rats with blood glucose < 250 mg/dL (still non-diabetic). After 1 week of recovery, rats were fasted for 12 h prior to the experiments, with free access to water. Animals were then anesthetized in an induction with isoflurane liquid for inhalation (Iso-Vet, Piramal Healthcare, UK) at a flow rate of 4–5 L/min together with 0.5–1 L/min of O_2_ and a midline laparotomy was performed to expose their jejunum for the following treatment administration. The HM-insulin-loaded mixed nanosystem (*n* = 8) at an HM-insulin dose of 100 IU/kg body weight and the same dose of blank nanosystem (*n* = 7) were intraduodenally administered in a maximum volume of 0.3 mL using a 25G needle. Rats were then allowed to completely recover and kept conscious, with free access to water, for the duration of the experiment. Additionally, 2 IU/kg body weight of an HM-insulin solution in saline were subcutaneously administered to a different control group (*n* = 6).

### Statistical analysis

GraphPad Prism 7 program (California, USA) was used to perform the statistical analysis. When applicable, data were compared using either the one-way or two-way ANOVA (specified in the corresponding figure caption according to the number of possible responsible factors for significant changes) followed by a Fisher’s LSD test, considering p-values lower than 0.05 as statistically significant.

## Results and discussion

The goal of this work was the rational design, development, and characterization of a nanoemulsion that fulfills the main requirements for an efficient oral peptide administration, namely, (i) possibility of producing a mono-dispersed population of carrier with nanometric size in a reproducible way, (ii) efficient HM-insulin loading, (iii) colloidal stability in simulated intestinal fluids and appropriate miscibility with them, (iv) mucodiffusive properties, (v) ability to interact with the intestinal cells without causing cytotoxic effects, and (vi) ability to lead an adequate pharmacological response once administered in vivo.

Miglyol® 812N, a medium chain caprylic/capric triglyceride with permeation enhancing properties was selected as the lipid core of the NE to entrap HM-insulin [43, 44]. MPEG-2000-DSPE sodium salt and poloxamer 407 were selected as surfactants not only for their colloidal stabilizing properties but also for their capacity to prevent the attachment of degrading enzymes onto the nanosystem [24, 45–48]. On the other hand, the presence of PEG molecules on the surface of the nanostructure was expected to promote its mucodiffusion [24, 48–52]. Sodium taurocholate (STC) was selected as an additional surfactant due to its penetration enhancing properties [53, 54]. Apart from the previously mentioned properties of MPEG-2000-DSPE and sodium taurocholate (STC), they are both negatively charged (Online resource 4), a fact that was supposed to favor the retention of the HM-insulin in the lipid core through the formation of hydrophobic ionic pairs [55–60]. Finally, using the HM-insulin, we expected that its hydrophobic domains (GRAVY ≈ − 0.0333) would favor its entrapment within the nanoemulsion (hydrophobic modifications illustrated in Online resource 5).

### Physicochemical, morphological, and drug loading properties of HM-insulin-loaded nanosystem

**Fig. 1:**
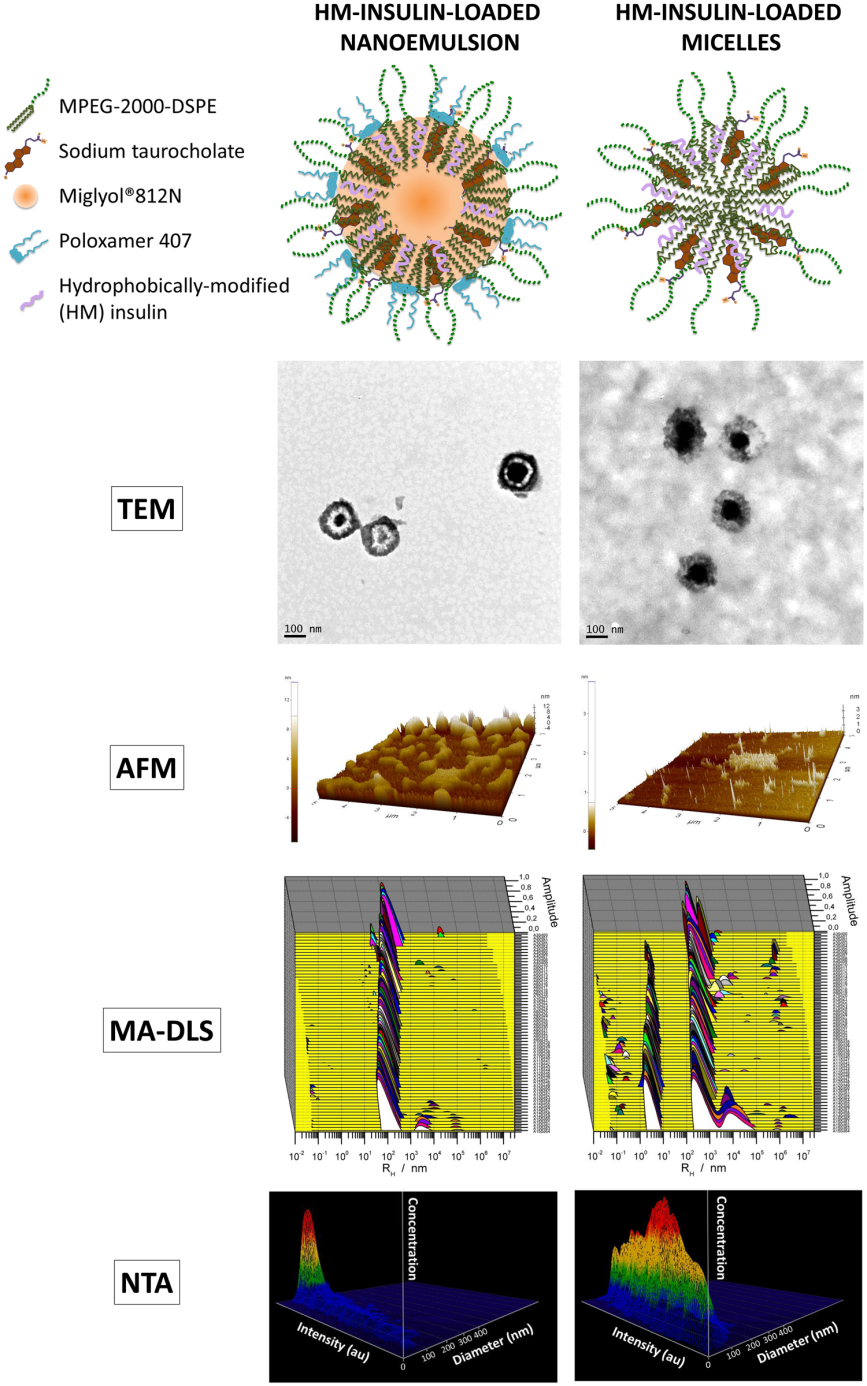
Schematic representation, TEM, AFM, MA-DLS, and NTA characterization of the separated species of the mixed nanosystem: HM-insulin-loaded NE (left) and HM-insulin-loaded micelles (right)

A schematic representation of the organization of the rationally selected components, leading to different nanostructures is illustrated in Fig. 1.

In the first step, an exploratory formulation study was performed in order to determine the amounts of the different components that were necessary to obtain a reproducible system with the desired properties. The physicochemical characterization of the selected composition led to the conclusion that it consisted of a mixture of NE and micelles (mixed nanosystem). We speculated that the presence of two saturated 18-carbon acyl chains in the MPEG-2000-DSPE molecules might have driven the self-assembling process and the formation of micelles through strong hydrophobic forces (low critical micelle concentration ≈ 1 × 10^−6^ M) [61]. Table 1 shows the data obtained for the mixed system and also for its two populations: nanoemulsion and micelles. The systems loaded with HM-insulin (in a free form or fluorescently labelled with FITC) and also co-encapsulated with the fluorescent marker DiD were characterized. The loading of these fluorescent markers was necessary for the subsequent in vitro characterization studies. Infrared spectroscopy of free FITC and HM-insulin and fluorescently labeled FITC-HM-insulin proved that FITC was covalently linked to HM-insulin (Online resource 6).

**Table 1:** Physicochemical properties and association efficiency (AE %) of the mixed nanosystem and its separate species (nanoemulsion and micelles), loaded with HM-insulin and DiD or 5-FITC fluorescent dyes (mean ± SD, n ≥ 3)

System	Loaded molecule(s)	Size (nm)	PDI	Ζ-potential (mV)	AE (%)
Mixed system (NE + micelles)	HM-insulin	219 ± 7	0.1	− 20 ± 1	n.d
	DiD/HM-insulin	217 ± 7	0.1	− 17 ± 1	n.d
	FITC-HM-insulin	214 ± 10	0.1	− 19 ± 1	n.d
NE	HM-insulin	216 ± 3	0.1	− 19 ± 3	46 ± 11
	DiD/HM-insulin	215 ± 12	0.1	− 15 ± 1	53 ± 1
	FITC-HM-insulin	216 ± 13	0.1	− 22 ± 1	48 ± 5
Micelles	HM-insulin	184 ± 4	0.1	− 16 ± 3	58 ± 9
	DiD/HM-insulin	181 ± 32	0.2	− 17 ± 2	60 ± 2
	FITC-HM-insulin	179 ± 18	0.1	− 15 ± 1	67 ± 1

Overall, the results in Table 1 indicate that the introduction of fluorescent tags did not significantly influence the size, PDI and ζ-potential of the formulations. The low negative surface charge of the system, which is attributed to the negatively charged lipids and surfactants, partially masked by the presence of PEG, is expected to contribute to stabilize the formulation in the intestinal fluids [24, 48, 62], and to facilitate its mucodiffusion [24, 48, 49].

For the determination of the HM-insulin association efficiency, the two populations were separated and the percentage of HM-insulin entrapped in each population was calculated. The results showed that HM-insulin was partitioned between the micelles and the droplets of the emulsion, resulting in total encapsulation values close to 100%. This corresponds to a final loading capacity (LC) of 1.65 ± 0.3% (w/w), being around 45% of the HM-insulin associated to the NE (LC = 1.13 ± 0.4%) and 55% associated to micelles (LC = 2.11 ± 0.4%).

In order to rigorously characterize the HM-insulin-loaded mixed nanosystem, the particle size of the separate species was also measured using multi-angle dynamic light scattering (MA-DLS) and nanoparticle tracking analysis (NTA) (Fig. 1). Interestingly, MA-DLS measurements showed a single population around 150 nm in the case of the HM-insulin-loaded NE, while it showed two differentiated populations for the HM-insulin-loaded micelles. Additionally, the analysis performed to blank micelles (Online resource 7) led us to conclude that both populations corresponded to one portion of non-loaded phospholipid micelles (the smallest population on the left) and another one of HM-insulin-loaded micelles (the biggest population on the right). These data supported the existence of an interaction between the peptide and the phospholipids strong enough to change the structural configuration of the micelles leading to the formation of a homogeneous population of HM-insulin-loaded micelles. Although the two species showed similar size, their different refractive index [63] was reflected in a distinct ability to scatter the light (represented along the intensity axe on the NTA 3D plot, Fig. 1). In addition, NTA analysis gave us the concentration of the two species in the mixed nanosystem, this being 2.81 × 10^12^ ± 1.84 × 10^11^ oily droplets/mL and 6.64 × 10^10^ ± 4.45 × 10^9^ micelles/mL (Fig. 1).

The size and shape of the separate species of the mixed nanosystem was further confirmed by TEM and AFM analysis. The images presented in Fig. 1 illustrate the different appearance of both species (NE and micelles).

### Stability of the HM-insulin-loaded mixed nanosystem during storage

The results of the colloidal stability studies during storage indicated that the system was stable for, at least, 6 months at 4 °C, room temperature (RT ≈ 25 °C) and 40 °C (Online resource 8). Size, PDI, and ζ-potential were monitored and found to be constant during this period. Furthermore, the HM-insulin content in the system was maintained for at least 3 months upon storage at 4 °C (no test was performed at other temperature).

### Interaction of the HM-insulin-loaded mixed nanosystem with bio-relevant media: miscibility, colloidal stability, and HM-insulin release

In the first step, the miscibility of the HM-insulin-loaded mixed nanosystem with the intestinal medium was assessed visually (Online resource 9). Then, the colloidal stability of the system upon incubation in simulated gastric and intestinal fluids at 37 °C was monitored by DLS (size, PDI, and derived count rate). The fluids selected were as follows: FaSSGF (fasted state simulated gastric fluid), SIF (simulated intestinal fluid) with and without 1% pancreatin, FaSSIF-V2 (fasted state simulated intestinal fluid version 2), and FeSSIF-V2 (fed state simulated intestinal fluid version 2) without enzymes (composition detailed in Online resource 2)*.* The results showed that the system remained colloidally stable for, at least, 4 h in all the biologically relevant media tested, independently of their composition (Online resource 10). This stability profile should be attributed to the protective steric layer conferred by the combination of MPEG-2000-DSPE and poloxamer 407 onto the oil/water interface [24, 47, 48, 62, 64, 65].

The assessment of the HM-insulin release in SIF medium required the use of high ultracentrifugation forces (84,035*g*). The disassembling of the system caused by these forces together with the high ionic strength of the release medium, caused the precipitation of 80% of the HM-insulin. The drastic conditions used in this study did not allow us to have reliable data about the HM-insulin release in these simulated fluids. However, we could speculate that the in vivo release of the associated HM-insulin will be probably driven by the disruption of the nanostructures.

### Assessment of the mucodiffusive behavior of the HM-insulin formulated in the mixed nanosystem (NE + micelles) and its separate species (MTA)

Once the nanosystem reaches the intestine, its proper diffusion through the intestinal mucus blanket is a crucial step before reaching the epithelium. For this purpose, the mucodiffusion capacity of the HM-insulin-loaded mixed nanosystem was evaluated by multiple particle tracking analysis using intestinal porcine mucus as mucus model [66]. This technique allows the estimation of the effective diffusion coefficient (*D*_*e*_) of each individual particle of the formulation by correlating its mean square displacement 〈*MSD*⟩with the time, according to the following equation (Eq. 3):

3 $${\langle MSD \rangle}=4{D}_{e}{\tau }^{\alpha }$$

where *α* gives information about the nature of the diffusion mode of the nanoparticle in mucus, *τ* is the time used for calculating their displacement in mucus, and *D*_*e*_ is the effective diffusion coefficient [24, 32–34].

**Fig. 2:**
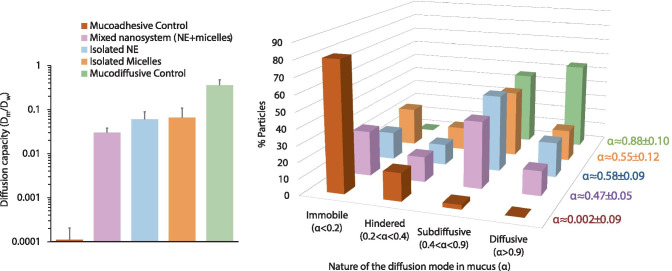
Diffusion capacity of the HM-insulin-loaded mixed nanosystem and its separate species calculated as mean effective diffusion coefficient in porcine mucus/mean effective diffusion coefficient in water (D_m_/D_w_) (left) and percentage of particles showing different α values (gives information about the nature of the diffusion mode in the corresponding matrix) (right). Mean ± SD; number of batches analyzed *n* = 3; *n* ≥ 1000 nanoparticles

Polystyrene nanoparticles and poloxamer 407-coated polystyrene nanoparticles were used as mucoadhesive and mucodiffusive controls. The diffusion capacities (D_m_/D_w_), calculated by dividing the mean effective diffusion coefficient of the particles in mucus (D_m_) vs the same in water (D_w_) at a 1 s time scale (τ) are represented in Fig. 2, left. Overall, the results showed that both the mixed nanosystem and its separate species exhibited an acceptable diffusion in mucus. The D_m_/D_w_ obtained were ≈ 4.2 × 10^−2^, 1.0 × 10^−1^, and 1.2 × 10^−1^ for the mixed nanosystem, the isolated NE, and the isolated micelles, respectively. The fact that HM-insulin-loaded mixed nanosystem displayed the lowest D_m_/D_w_ ratio (≈ 4.2 × 10^−2^) could be attributed to a higher concentration of particles in suspension due to the coexistence of both species. In the same line, the mean α values were in the 0.47–0.58 range, which is an indicative of a close to free diffusion mode of these nanostructures in mucus. One of the main strengths of particle tracking relies on its potential for the analysis of the individual trajectories of a formulation. In this regard, in order to deeply characterize the diffusion mode of these nanostructures in mucus, the population distribution of the α values of each system was analyzed in detail (Fig. 2, right). For this purpose, four different α populations were set as follows: α ≥ 0.9 (diffusive particles), 0.4 ≤ α < 0.9 (subdiffusive particles), 0.2 ≤ α < 0.4 (hindered subdiffusive particles), and 0.2 < α (immobile particles) [32, 34, 35]. The results showed that mucoadhesive control nanoparticles were immobile or displayed hindered diffusion, whereas the mucodiffusive control showed a subdiffusive/diffusive behavior. Similar diffusion was observed for both the mixed nanosystem and the individual NE or micelles, showing around 30–40% of immobile/hindered species while 60–70% of particles displayed diffusive or subdiffusive behavior. These results suggest that, despite the fact that the hydrophobic character of the NE and micelles could promote their retention into the mucus matrix [62, 67], the presence of PEG derivatives with a PEG-Mw below 10 kDa on the surface was enough to enhance their mucodiffusion capacity [24, 48, 51, 67].

### In vitro cell culture studies of HM-insulin formulated in the mixed nanosystem

#### Colloidal stability and HM-insulin release in cell culture media

The colloidal stability of the HM-insulin-loaded mixed nanosystem upon incubation in cell culture media (HBSS, supplemented-DMEM and supplemented-EMEM) for up to 24 h was monitored by DLS (size, PDI, and derived count rate). Results showed that the system remained colloidally stable for, at least, 24 h in all cellular media tested, independently of their composition (Online resource 11). The potential release of HM-insulin in the cell culture medium (HBSS and supplemented-EMEM) was also quantified. The results showed negligible HM-insulin release after 4 h incubation with HBSS (*n* = 3), whereas 86 ± 1% of the HM-insulin was released from the mixed nanosystem (*n* = 3) after 20 h contact with supplemented-EMEM.

### Cytotoxicity studies of both blank and HM-insulin-loaded nanosystem, in Caco-2, C2BBe1, and HT29-MTX cells (ATP, NRU, LDH, and MTT)

Although the Caco-2 cell line has been widely used as an in vitro model to study the interaction of the formulations with the enterocytes [36, 68, 69], nowadays it is considered that the presence of mucus-secreting goblet cells is relevant in order to mimic the barrier function of the intestinal epithelium [70]. Hence, the cytotoxicity of the nanosystem was first evaluated in Caco-2 cells and then in both the C2BBe1 human colon carcinoma Caco-2 clone (morphologically more homogeneous than Caco-2 cells, with the microvilliar brush border exclusively localized in the apical side) and mucus-secreting HT29-MTX (smaller microvilli, and softer tight junctions than Caco-2 cells) by MTT [30, 68, 71–73].

**Fig. 3:**
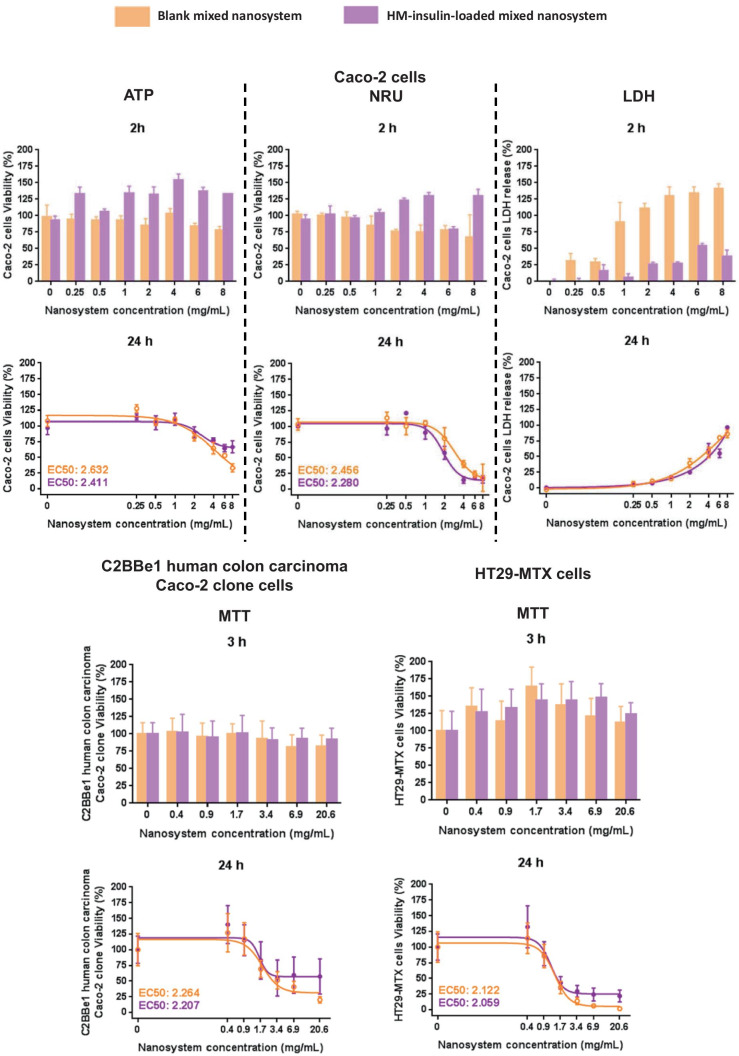
Upper panel: Caco- 2 cell viability (%) (ATP and NRU) and plasma membrane damage (% LDH release) after 2 (bar charts) and 24 h (line charts) incubation with different concentrations of blank and HM-insulin-loaded mixed nanosystems. Lower panel: cell viability (%) (MTT) of C2BBe1 human colon carcinoma Caco-2 clone (left) and HT29-MTX (right) cells after 3 (bar charts) and 24 h (line charts) incubation with different concentrations of blank and HM-insulin-loaded mixed nanosystems. Mean ± SD, *n* ≥ 3

The cytotoxicity of the blank and HM-insulin-loaded mixed nanosystem was assessed in Caco-2 cells using three different techniques (ATP, NRU, and LDH). While non-important cytotoxic effects were found after a 2 h incubation time, for concentrations as high as 8 mg/mL, a noticeable dose-dependent LDH release was observed (Fig. 3, bar charts upper panel). A slightly higher cell viability was observed after treating the cells with the HM-insulin-loaded mixed nanosystem (ATP and NRU assays), which could be related to the cell proliferation properties of insulin [74–76]. Accordingly, higher LDH release was detected in cells exposed to concentrations of blank nanosystem > 1 mg/mL. These results are in the same line than those reported for similar lipid-based nanosystems [20, 30, 77].

After a 24 h incubation time, a dose-dependent cytotoxic profile, probably attributable to the penetration enhancing properties of the formulation components and their interaction with the enterocytes [43, 53, 78–80], was found in the three assays (Fig. 3, line charts upper panel). EC50 estimated values were similar from both the blank and the HM-insulin-loaded nanosystem, ranging approximately from 2.2 to 2.6 mg/mL. Overall, these values are an indication of the very low toxicity of the nanosystem.

Additionally, the cytotoxicity was assessed in C2BBe1 human colon carcinoma Caco-2 clone and mucus secreting HT29-MTX after 3 and 24 h incubation. The results were in the same line than those obtained from the first screening performed in Caco-2 cells, confirming that non-cytotoxic effects were caused after 3 h incubation in both C2BBe1 human colon carcinoma Caco-2 clone and HT29-MTX cells for both blank and HM-insulin-loaded mixed nanosystem (Fig. 3, bar charts lower panel), whereas a dose-dependent cytotoxic effect was observed after 24 h incubation with estimated EC50 ranged from 2.0 to 2.3 mg/mL of system concentration (Fig. 3, line charts lower panel).

#### Interaction of FITC-HM-insulin-loaded mixed nanosystem with Caco-2 and C2BBe1 cell mono-cultures and with C2BBe1/HT29-MTX co-culture

**Fig. 4:**
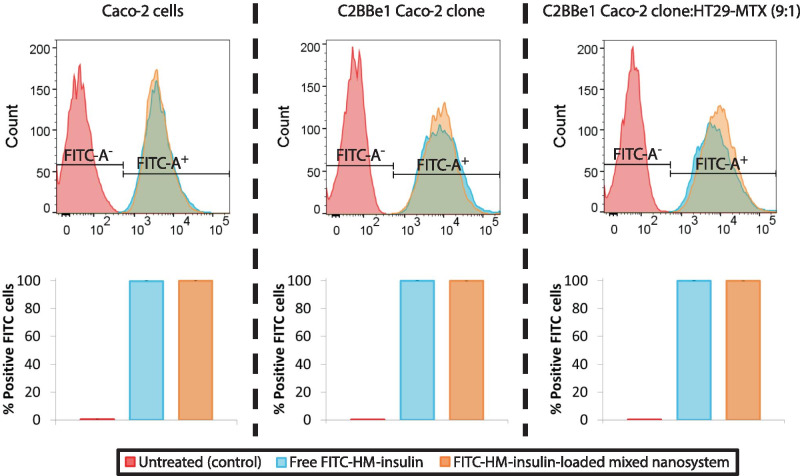
Upper panel: flow cytometry profile showing the interaction of free FITC-HM-insulin (blue) and FITC-HM-insulin-loaded mixed system (NE and micelles) (orange) with Caco-2 (left) and C2BBe1 human colon carcinoma Caco-2 clone (middle) monocultures, as well as with C2BBe1 human colon carcinoma Caco-2 clone:HT29-MTX (9:1) co-culture (right) compared to the non-treated control cells (red). Lower panel: percentage of FITC-positive cells calculated based on parent cells in Caco-2 (left) and C2BBe1 human colon carcinoma Caco-2 clone (middle) monocultures, as well as with C2BBe1 human colon carcinoma Caco-2 clone:HT29-MTX (9:1) co-culture (right). Mean ± SD, *n* ≥ 3 (two-way ANOVA followed by a Fisher’s LSD test were applied for the statistical analysis; significance level comparing to the control ****p ≤ 0.001)

The interaction of both free FITC-HM-insulin and FITC-HM-insulin-loaded mixed nanosystem with Caco-2 and C2BBe1 human colon carcinoma Caco-2 clone monocultures, as well as with C2BBe1 human colon carcinoma Caco-2 clone:HT29-MTX (9:1) co-culture was quantitatively analyzed using FACS. Untreated cells were used as controls. Measurements were done after 3 h incubation for a FITC-HM-insulin concentration of 45 µg/mL, corresponding to 3 mg/mL of nanosystem (non-cytotoxic dose, see “Cytotoxicity studies of both blank and HM-insulin-loaded nanosystem in Caco-2, C2BBe1, and HT29-MTX cells (ATP, NRU, LDH, and MTT)” section*.*). FACS showed significant differences (*p* ≤ 0.0001) between the non-treated cells and those treated with free FITC-HM-insulin or FITC-HM-insulin-loaded mixed nanosystem, which were 100% fluorescence positive irrespective of the cell line (Fig. 4). These results indicate that both free HM-insulin and HM-insulin-loaded mixed nanosystem had the capacity to interact with the intestinal cells (≈ 100% of FITC-positive cells). The higher cellular interaction found for the free HM-insulin in comparison with the usually observed for the regular insulin, was attributable to an improved cell membrane interaction driven by the hydrophobic modifications of the peptide.

**Fig. 5:**
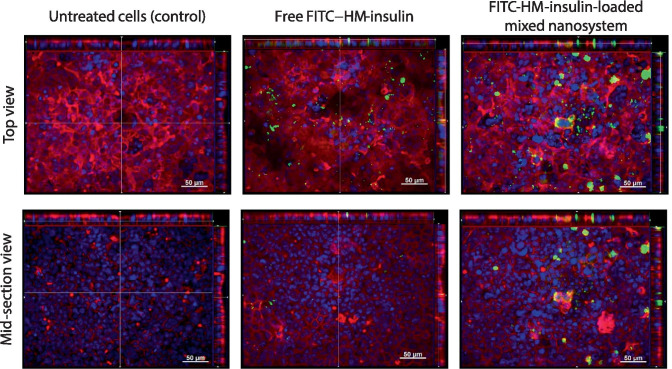
Confocal images in Caco-2 monolayers (top and mid-section views at × 25) representing the y-z, x-z, and x-y view of Caco-2 monolayers after 2 h incubation with free FITC-HM-insulin (middle) and FITC-HM-insulin-loaded mixed nanosystem (NE + micelles) (right). Untreated monolayers were used as control (left). Cell membranes (rhodamine–phalloidin), cell nuclei (DAPI), and HM-insulin (FITC) are visualized in red, blue, and green, respectively

The interaction of the nanosystems with the monolayers was also analyzed using confocal laser scanning microscopy. For this purpose, since no differences were found in FACS for the different cell types, confocal images were taken in Caco-2 monolayers after 2 h incubation with both free FITC-HM-insulin and FITC-HM-insulin-loaded mixed nanosystem at the same concentration used for FACS. Images of untreated monolayers were taken as a control. Interestingly, as shown in Fig. 5, the confocal studies showed differences regarding the type of interactions displayed by the free FITC-HM-insulin and FITC-HM-insulin-loaded mixed nanosystem, especially in the X–Z profile and in the mid-section of X–Y view. While almost no FITC-HM-insulin was found in the X–Z profile of the monolayer, FITC-HM-insulin-loaded mixed nanosystem appeared also located inside the cells. This internalization of the system could be attributed to the permeation enhancing properties of some components of the formulation, such as oil and surfactants, and the special properties of the nanostructures by itself.

On the other hand, TEER analysis performed before and 2 h after the experiment confirmed that neither free FITC-HM-insulin nor FITC-HM-insulin-loaded mixed nanosystem caused a significant effect in the TEER values respect to the control (untreated cells) (Online resource 12). These results indicate that the tight junctions were not affected by either treatment, thus discarding the possibility of paracellular FITC-HM-insulin transport.

#### Bioactivity of HM-insulin formulated in the mixed nanosystem

**Fig. 6:**
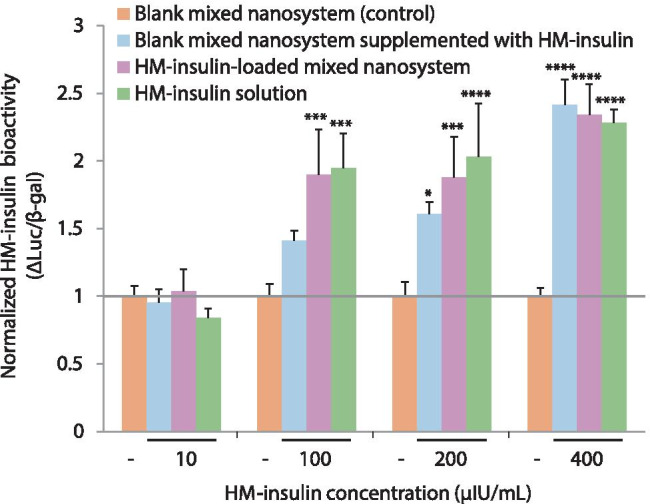
HM-insulin activity in human liver cells using the insulin target-gene (HMG-CoA synthase) promoter assay. Normalized HM-insulin bioactivity (∆Luc/β-gal) obtained after HEP  G2 incubation with increasing HM-insulin concentrations (from 10 to 400 µIU/mL) in form of blank mixed nanosystem supplemented with HM-insulin (blue), HM-insulin-loaded mixed nanosystem (purple), and HM-insulin solution (green). Blank mixed nanosystem was used as negative control (orange). Mean ± SD, *n* = 5 independent experiments with 6–8 replicates per condition in each (two-way ANOVA followed by a Fisher’s LSD test were applied for the statistical analysis; significance levels comparing to the control *p ≤ 0.05; ***p ≤ 0.001, ****p ≤ 0.001)

Since it is well known that peptides are labile macromolecules that may be inactivated as a consequence of the formulation process [81], the preservation of the HM-insulin bioactivity after its inclusion in the nanosystem was first studied in vitro in Hep G2 cells (enriched in insulin receptor expression). This assay involved the transfection of one promoter plasmid (pSynSRE‐T‐luc, promoter activation of an insulin early target gene) and one control plasmid (pRSV‐βgal, for transfection control). Different concentrations of the HM-insulin-loaded nanosystem were assayed using blank mixed nanosystem and HM-insulin solutions as negative and positive controls respectively. Additionally, different concentrations of the blank mixed nanosystem supplemented with HM-insulin were included in the study to discard any potential interference (either inhibition or promotion of the insulin bioactivity) of the formulation components with the assay. The intensity ratio between luciferase (promoter mediated) and β‐galactosidase (control) expressions was calculated, and results were normalized respect to the negative control (blank mixed nanosystem) and represented as increment ∆Luc/β-gal in Fig. 6. Results indicated that the HM-insulin associated to the mixed nanosystem, and consequently released from it in the course of the experiment, exhibited the same activity than both free HM-insulin alone and blank mixed nanosystem later spiked with HM-insulin (no significant differences at any of the concentrations tested). Therefore, it can be concluded that the bioactivity of HM-insulin was preserved following its incorporation into the mixed nanosystem and its subsequent release.

### In vivo hypoglycemic response of HM-insulin formulated in the mixed nanosystem

The evaluation of the hypoglycemic response of HM-insulin-loaded formulations has been performed in healthy [54, 82] and diabetic [21, 30, 54, 83] animal models. In normoglycemic rats, the exogenous insulin may decrease the secretion of the endogenous one by the β-cells and its effect might be hindered by the autoregulation phenomenon [18, 84]. On the other hand, the commonly used streptozocine (STZ) model leads to different degrees of β-cell deficiency and, hence, very variable glycemic responses [21, 30].

#### Healthy rats

##### HM-insulin dose–response and bioactivity after its inclusion in the nanosystem following subcutaneous (SC) administration to healthy rats

**Fig. 7:**
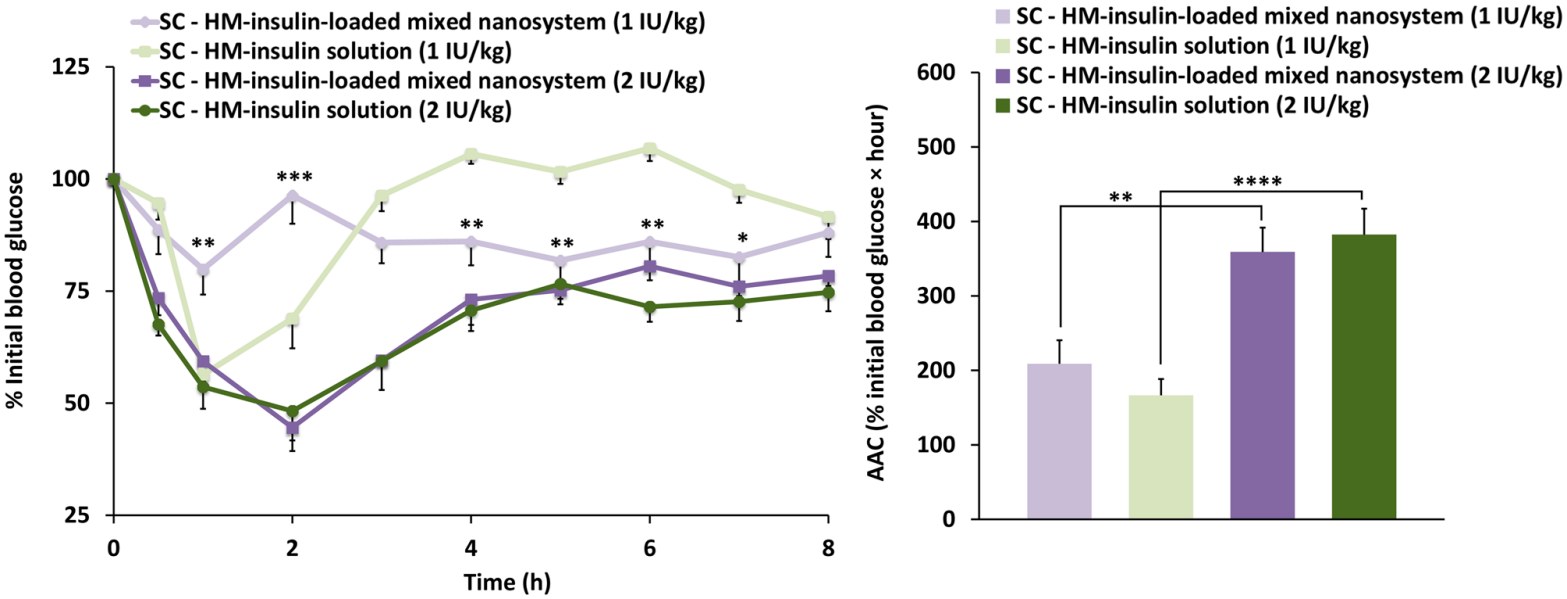
Left: Normalized hypoglycemic effect (% of the initial blood glucose) following subcutaneous (SC) administration of a saline HM-insulin solution and HM-insulin-loaded mixed nanosystem (NE + micelles) at both 1 (*n* = 8) and 2 IU/kg body weight (*n* = 13) to healthy rats (mean ± SEM) (two-way ANOVA followed by a Fisher’s LSD test were applied for the statistical analysis; significance levels comparing to the HM-insulin solution at the same concentration *p ≤ 0.05; **p ≤ 0.01; ***p ≤ 0.005). Right: Area above the curve at time = 8 h calculated by establishing 120% as upper limit (% initial blood glucose × hour) (mean ± SEM) (one-way ANOVA followed by a Fisher’s LSD test were applied for the statistical analysis; significance levels **p ≤ 0.01; ****p ≤ 0.0001)

We first conducted a dose–response study (1 and 2 IU/kg body weight) following subcutaneous administration (SC) of HM-insulin in the free form or associated to the nanosystem to fasted healthy rats. Figure 7 shows the glucose values normalized respect to the glucose baseline level (measured 30 min prior to the treatments administration), which was considered as 100%.

The results indicated that the overall hypoglycemic response measured by means of the area above the curve (AAC) was similar for the free and encapsulated HM-insulin; however, it was significantly influenced by the dose (Fig. 7, right). Beyond these similar global values, the profiles observed for the two HM-insulin doses were different (Fig. 7, left). In the case of the low dose (1 IU/kg), HM-insulin-loaded nanosystem led to a low and prolonged hypoglycemic response as compared to the profile observed for the high dose (2 IU/kg). The reason why this delayed response was not observed for the high HM-insulin dose remains to be elucidated. Irrespective of this, these results corroborated that HM-insulin preserves its bioactivity in vivo after being incorporated in the formulation.

##### Effect on blood glucose levels following intrajejunal (IJ) administration of 100 IU/kg of HM-insulin-loaded mixed nanosystem to healthy rats

Firstly, we performed a preliminary in vivo study aimed at exploring the influence of the site of administration, intraduodenal vs. intrajejunal on the hypoglycemic effect. As expected [30], the results indicated that there were no differences in the glucose levels following both modalities of administration (data not shown).

**Fig. 8:**
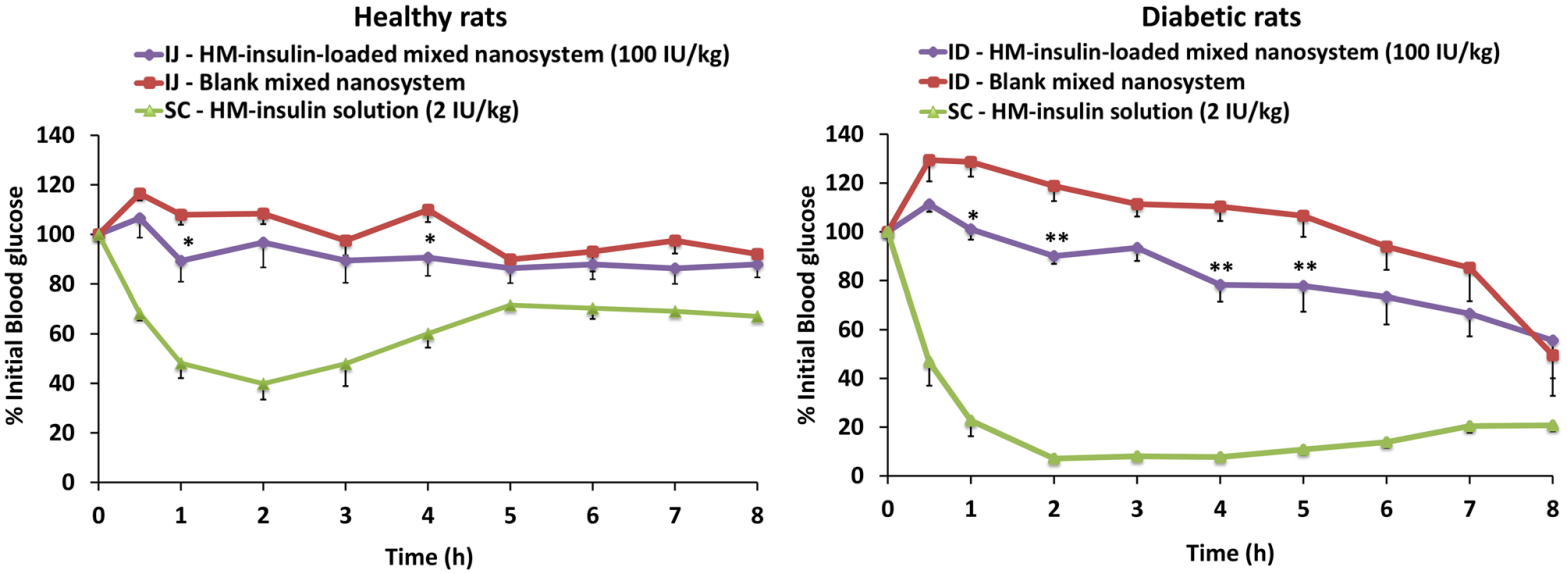
Normalized hypoglycemic effect (% of the initial blood glucose) following either intrajejunal (IJ) or intraduodenal (ID) administration of HM-insulin-loaded mixed nanosystem (*n* = 8) at 100 IU/kg, blank mixed nanosystem (*n* = 6) and subcutaneous (SC) administration of a HM-insulin solution (*n* = 6) at 2 IU/kg to healthy (left) and diabetic (right) rats (mean ± SEM) (two-way ANOVA followed by a Fisher’s LSD test were applied for the statistical analysis; significance levels for HM-insulin-loaded mixed nanosystem comparing to the blank nanosystem *p ≤ 0.05; **p ≤ 0.01)

Based on these preliminary results and the dose–response effect previously observed, the HM-insulin-loaded mixed nanosystem was intrajejunally administered (IJ) (100 IU/kg body weight) to healthy rats, using the blank mixed nanosystem (IJ) and a saline HM-insulin solution (SC) (2 IU/kg body weight) as negative and positive controls, respectively. Figure 8, left, shows the blood glucose levels monitored for up to 8 h post-administration and normalized respect to the glucose baseline level (measured 30 min prior to the treatments administration). A slight hypoglycemic effect, which was significant different (*p* ≤ 0.05) respect to the control (≈ 20%), was observed at 1 and 4 h post-administration. These results are comparable to those previously reported and performed under similar conditions [21, 30, 54, 83], although higher hypoglycemic responses have also been reported for non-modified insulin under different experimental conditions (i.e., intraileal administration, long fasting period, anesthesia, glucose exogenously overload) [18, 84–87]. The conclusion from the analysis of these data and those previously reported is that the experimental conditions may significantly affect the intensity of the hypoglycemic response.

#### Diabetic rats

##### Effect on blood glucose levels following intraduodenal (ID) administration of 100 IU/kg of HM-insulin-loaded mixed nanosystem to diabetic rats

The efficacy of the formulation was also evaluated in a diabetic rat model [21, 30, 54, 83]. This animal model was selected apart from its physiological relevance, to avoid the possible interferences of the autoregulation phenomenon, which may suppress the secretion of endogenous insulin by the β-cell in healthy animals [18, 84]. For this purpose, and based on the dose–response curves previously obtained, the HM-insulin-loaded mixed nanosystem was intraduodenally administered (ID) (100 IU/kg body weight) to diabetic rats, using the blank mixed nanosystem (ID) and a saline HM-insulin solution (SC) (2 IU/kg body weight) as negative and positive controls, respectively. Blood glucose levels were monitored up to 8 h post-administration and values were normalized respect to the glucose baseline level (measured 30 min prior to the treatments administration) considered as 100%.

The results presented in Fig. 8, right, indicated that the intraduodenal administration of the HM-insulin-loaded mixed nanosystem triggered a significant hypoglycemic response (≈ 30%) compared to the control (intradudenally administered blank mixed nanosystem) at 1 (*p* ≤ 0.05), 2, 4, and 5 (*p* ≤ 0.01) h post-administration.

Overall, the results suggest that the mixed nanosystem was able to protect a portion of the HM-insulin intraduodenally administered from the degradation and allowed its intestinal absorption, triggering, subsequently, a significant hypoglycemic response (*p* ≤ 0.01). However, bearing in mind the rational design of this formulation, and its favorable properties displayed in vitro, we must admit that its performance was lower than expected, a result that brings the difficult in vitro–in vivo correlation to light.

In this regard, it is important to highlight that the majority of the literature dealing with oral insulin delivery carriers does not report the results of the in vivo administration, where presumably conditions are slightly harsher than in vitro. This limited information raises the question about whether these previous reports consciously omit the in vivo work or lack of such information. However, when the information is available, the differences in the followed in vivo experimental protocols make the comparison among the different works difficult. The majority of the studies were performed in diabetic rats, and the data reported do not generally provide a good understanding of the variable responses obtained. This might be related to the commonly used streptozocine model, which usually leads to different degrees of β-cell deficiency and, hence, very variable glycemic responses. These facts, led us to emphasize the importance of standardizing experimental protocols to obtain more predictive values in vitro–in vivo to facilitate the clinical translation of the research made in this field.

## Conclusions

Herein, a new formulation consisting of a mixture of nanoemulsion and micelles was designed, developed, and fully characterized, by rationally selecting biomaterials with stabilizing, penetration and mucodiffusive properties. A hydrophobically modified insulin was used as model peptide to assess the ability of the nanosystem to successfully deliver peptides orally. The HM-loaded mixed nanosystem exhibited in vitro appropriate properties, such as good stability, mucodiffusion, cell interaction, and uptake without cytotoxic effects, which reinforced the interest of its further in vivo evaluation. Following its intra-intestinal administration in both healthy and diabetic rats, a significant, but moderate hypoglycemic response more noticeable in the diabetic model, was observed in vivo. Overall, despite the promising properties displayed by the formulation here disclosed and the significant effect observed in vivo, the in vitro–in vivo correlation when referring to the rational design of oral peptide delivery formulations remains still a challenge.

## Supplementary Information

Below is the link to the electronic supplementary material.

Supplementary file1 (PDF 873 KB)

## Data Availability

The datasets generated during this work can be available on reasonable request.
