# Correction to: A nanoemulsion/micelles mixed nanosystem for the oral administration of hydrophobically modified insulin

**DOI:** 10.1007/s13346-021-00957-y

**Published:** 2021-03-19

**Authors:** Irene Santalices, Carlos Vázquez‑Vázquez, Manuel J. Santander‑Ortega, Victoria Lozano, Francisca Araújo, Bruno Sarmento, Neha Shrestha, Veronique Préat, Miguel Chenlo, Clara V. Alvarez, Federico Benetti, Juan Cuñarro, Sulay Tovar, Dolores Torres, María José Alonso

**Affiliations:** 1 Center for Research in Molecular Medicine and Chronic Diseases (CIMUS), University of Santiago de Compostela, Campus Vida, 15782 Santiago de Compostela, Spain; 2 Department of Pharmaceutics and Pharmaceutical Technology, School of Pharmacy, University of Santiago de Compostela, Campus Vida, 15782 Santiago de Compostela, Spain; 3 Department of Physical Chemistry, Faculty of Chemistry, University of Santiago de Compostela, Campus Vida, 15782 Santiago de Compostela, Spain; 4 Cellular Neuroanatomy and Molecular Chemistry of Central Nervous System Group, School of Pharmacy, University of Castilla-La Mancha, 02071 Albacete, Spain; 5 Regional Centre of Biomedical Research (CRIB), University of Castilla-La Mancha, 02071 Albacete, Spain; 6 Instituto de Investigação E Inovação Em Saúde (i3S), Instituto Nacional de Engenharia Biomédica (INEB), Universidade Do Porto, Rua Alfredo Allen 208, 4200-135 Porto, Portugal; 7 Instituto de Investigacão E Formacão Avançada Em Ciências E Tecnologias da Saúde (CESPU), 4585-116 Gandra, Portugal; 8 Advanced Drug Delivery and Biomaterials, Université Catholique de Louvain, Louvain Drug Research Institute, 1200 Brussels, Belgium; 9 Neoplasia & Endocrine Differentiation Group, Center for Research in Molecular Medicine and Chronic Diseases (CIMUS), University of Santiago de Compostela, Campus Vida15782, Santiago de Compostela, Spain; 10 ECSIN-European Center for the Sustainable Impact of Nanotechnology, ECAMRICERT SRL, Padova, Italy

## Correction to: Drug Delivery and Translational Research 10.1007/s13346-021-00920-x

The article A nanoemulsion/micelles mixed nanosystem for the oral administration of hydrophobically modified insulin, written by Santalices et al., was originally published electronically on the publisher’s internet portal on February 11, 2021, without open access. With the authors' decision to opt for Open Choice the copyright of the article changed on February 25, 2021, to ©The Author(s) and the article is forthwith distributed under a Creative Commons Attribution 4.0 International License.

