# Supplementary material for: A nanoemulsion/micelles mixed nanosystem for the oral administration of hydrophobically modified insulin
Source: Drug Deliv Transl Res. 2021 Feb 11;11(2):524–45. doi: 10.1007/s13346-021-00920-x (PMC7987602; doi:10.1007/s13346-021-00920-x)
Supplement: Supplementary file 1 — Supplementary file1 (PDF 873 KB) [file 13346_2021_920_MOESM1_ESM.pdf]

Supplementary Information

Article title: A nanoemulsion/micelles mixed nanosystem for the oral administration of hydrophobically modified insulin

Journal name: Drug Delivery and Translational Research

Author names: *Irene Santalices[1,2], Carlos Vázquez-Vázquez[3], Manuel J. Santander-Ortega[4,5], Victoria Lozano[4,5], Francisca Araújo[6], Bruno Sarmento[6,7], Neha Shrestha[8], Veronique Préat[8], Miguel Chenlo[8], Clara V Alvarez[9], Federico Benetti[10], Juan Cuñarro[1], Sulay Tovar[1], Dolores Torres[2], María José Alonso[1,2*].*

[*]Corresponding author e-mail address: mariaj.alonso@usc.es

1. *Center for Research in Molecular Medicine and Chronic Diseases (CIMUS), Campus Vida, University of Santiago de Compostela, Santiago de Compostela 15782, Spain.*
2. *Department of Pharmaceutics and Pharmaceutical Technology, School of Pharmacy, Campus Vida, University of Santiago de Compostela, Santiago de Compostela 15782, Spain.*
3. *Department of Physical Chemistry, Faculty of Chemistry, Campus Vida, University of Santiago de Compostela, Santiago de Compostela 15782, Spain.*
4. *Cellular Neuroanatomy and Molecular Chemistry of Central Nervous System Group, School of Pharmacy, University of Castilla-La Mancha, Albacete 02071, Spain.*
5. *Regional Centre of Biomedical Research (CRIB), University of Castilla-La Mancha, Albacete 02071, Spain.*
6. *Instituto de Investigação e Inovação em Saúde (i3S) and Instituto Nacional de Engenharia Biomédica (INEB), Universidade do Porto, Rua Alfredo Allen 208, 4200-135 Porto, Portugal.*
7. *Instituto de Investigacão e Formacão Avançada em Ciências e Tecnologias da Saúde (CESPU), 4585-116 Gandra, Portugal*
8. *Université Catholique de Louvain, Louvain Drug Research Institute, Advanced Drug Delivery and Biomaterials, 1200 Brussels, Belgium*
9. *Neoplasia & Endocrine Differentiation group, Center for Research in Molecular Medicine and Chronic Diseases (CIMUS), Campus Vida, University of Santiago de Compostela, Santiago de Compostela 15782, Spain.*
10. *European Center for the Sustainable Impact of Nanotechnology (ECSIN), ECAMRICERT SRL, I-45100, Rovigo, Italy.*

Supplementary Material

Online Resource 1. *UPLC analytical method for HM-insulin quantification.*

Time (min)	Phase A (%)	Phase B (%)
0.00	85	15
0.20	85	15
3.54	50	50
3.78	10	90
4.49	10	90
4.53	85	15
5.43	85	15

Online Resource 2. *FaSSGF, SIF, FaSSIF−V2 and FeSSIF−V2 without enzymes media composition* [1,2].

Composition	FaSSGF without enzymes	SIF	FaSSIF−V2	FeSSIF−V2 without enzymes
Sodium hydroxide		15.4 mM	34.8 mM	81.65 mM
Monobasic potassium phosphate		50 mM		
Sodium taurocholate	0.08 mM		3 mM	10 mM
Lecithin	0.02 mM		0.2 mM	2 mM
Maleic acid			19.12 mM	55.02 mM
Glyceryl monooleate				5 mM
Sodium oleate				0.8 mM
Sodium chloride	34.2 mM		68.62 mM	125.5 mM
Calcium chloride				5 mM
pH	1.6 / 4	6.8	6.5	5.8

FASSGF: Fasted State Simulated Gastric Fluid ;SIF: Simulated Intestinal Fluid; FaSSIF-V2: Fasted State Simulated Intestinal Fluid Version 2; FeSSIF-V2: Fed State Simulated Intestinal Fluid Version 2.

Online Resource 3. *Luciferase Assay Buffer, Luciferin solution, Buffer Z and Buffer ONPG media composition* [3].

Composition	Luciferase Assay Buffer	Luciferin solution	Buffer Z	Buffer ONPG
Glycylglycine (pH 7.8)	25 mM	25 mM		
K_2PO_4	15 mM			
Mg_2SO_4	15 mM		1 mM	
Ethyleneglycoltetraacetic acid (EGTA)	4 mM			
ATP	2 mM			
Dithiothreitol (DTT)	1 mM			
D−Luciferin		140 µM		
Na_2HPO_4			60 mM	577 mM
$Na_2H_2PO_4$			40 mM	423 mM
KCl			10 mM	
β−mercaptoethanol (pH 7.5)			50 mM	
Orthonitrophenolgalactopyranoside (ONPG)				13.3 mM

Online Resource 4. *Chemical structure of STC and MPEG-2000-DSPE.*

STC

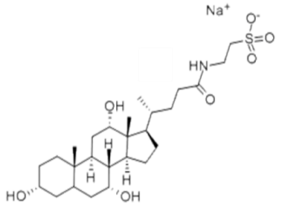

MPEG-2000-DSPE

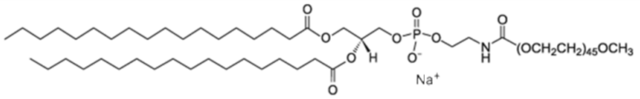

Online Resource 5. *Amino acid sequence of human insulin and HM-insulin, with the hydrophobic modifications represented in green.*

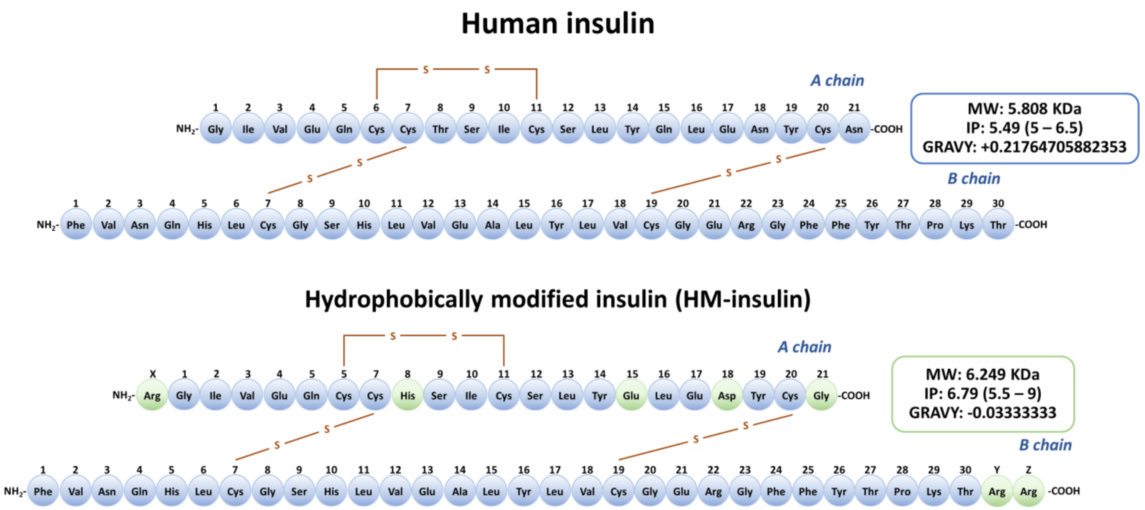

Online Resource 6. *Infrared spectroscopy spectra of FITC, HM-insulin and FITC-HM-insulin.*

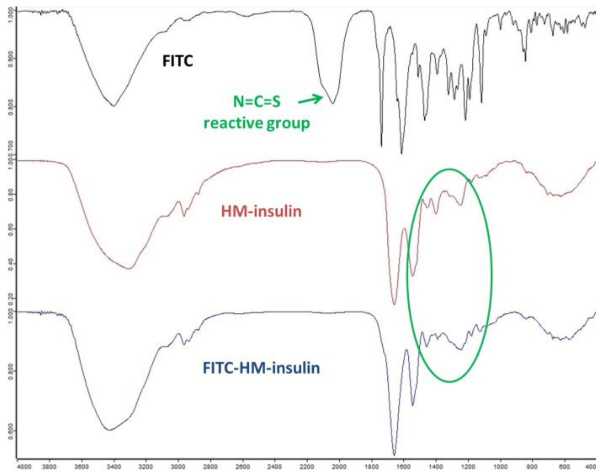

Online Resource 7. *TEM and MA-DLS characterization of the non-loaded micelles.*

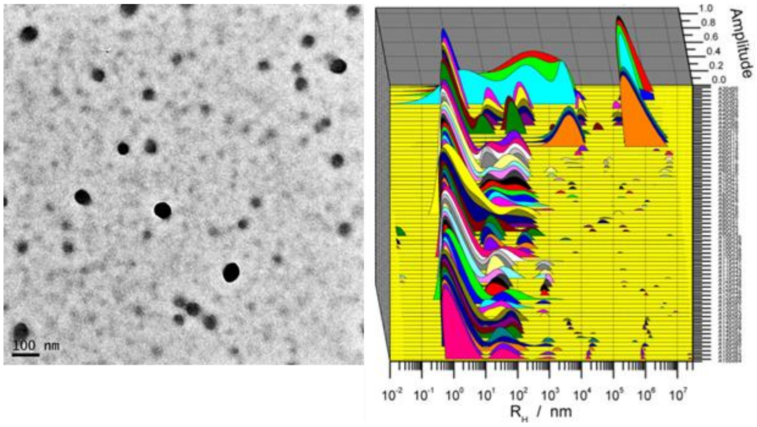

Online Resource 8. *Colloidal stability studies during storage of the HM-insulin-loaded mixed nanosystem (NE + micelles) at three different conditions: 4 °C (blue), RT ≈ 25 °C (green) and 40 °C (red). Mean ± SD, n ≥ 3.*

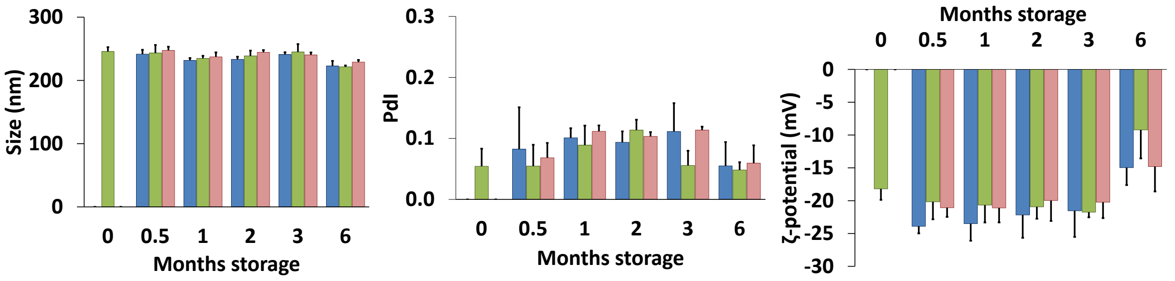

Online Resource 9. *Miscibility of the HM-insulin-loaded mixed nanosystem (NE + micelles) after its incubation in SIF.*

HM-insulin-loaded mixed nanosystem + SIF

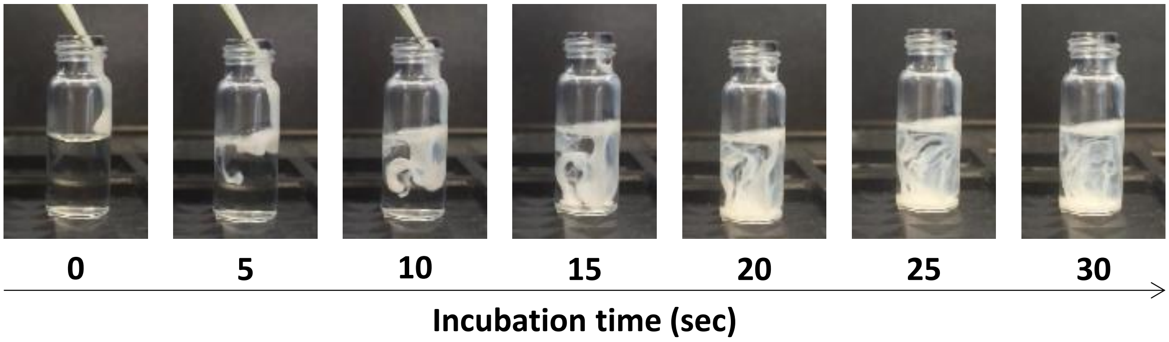

Online Resource 10. *Colloidal stability profile of the HM-insulin-loaded mixed nanosystem (NE + micelles) after its incubation in different simulated bio-relevant media (SGF, SIF, FaSSIF−v2, FeSSIF−v2 without pancreatin and 1 % pancreatin supplemented-SIF) at 37 °C. Mean ± SD, n ≥ 3.*

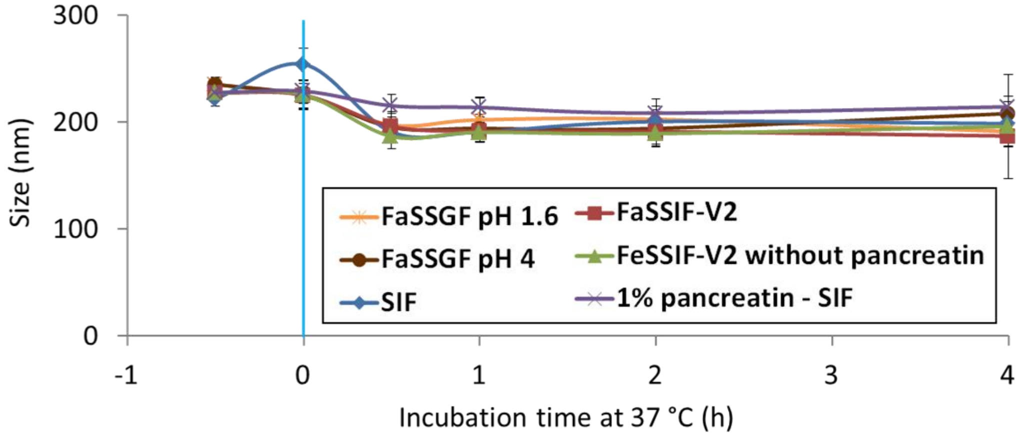

Online Resource 11. *Colloidal stability profile of the HM-insulin -loaded mixed nanosystem (NE + micelles) after its incubation in different cellular media (HBSS, supplemented-DMEM and supplemented EMEM) at 37 °C. Mean ± SD, n ≥ 3.*

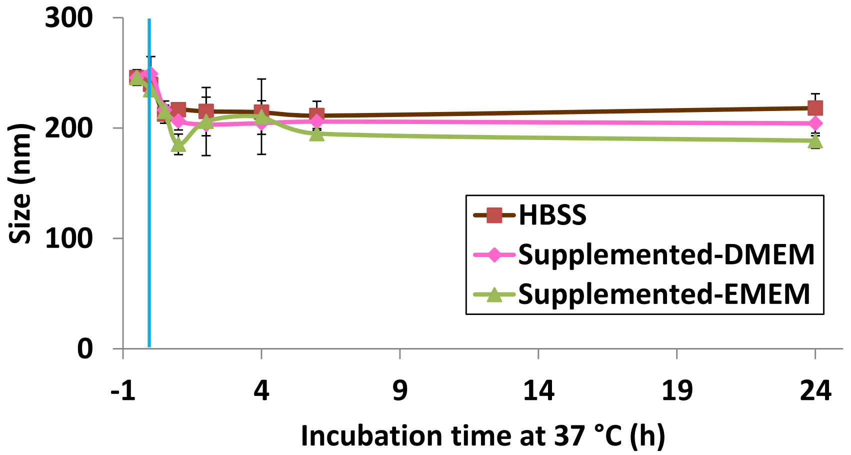

Online Resource 12. *Caco−2 cells TEER values normalized respect to control cells (red) before and after 2 hours incubation with either free FITC-HM-insulin (green) or FITC-HM-insulin-loaded nanosystem (NE + micelles) (purple) Mean ± SD, n ≥ 3 (Two-way ANOVA followed by a Fisher's LSD test were applied for the statistical analysis).*

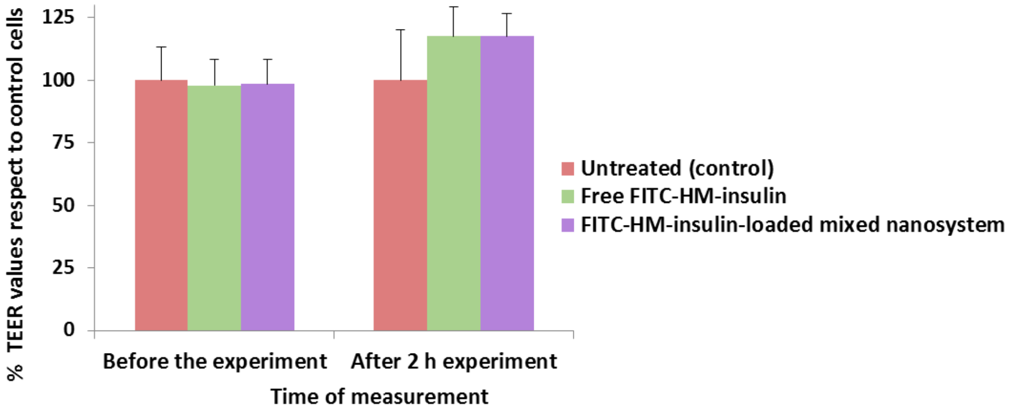

References

[1] Z. Niu, E. Tedesco, F. Benetti, A. Mabondzo, I.M. Montagner, I. Marigo, D. Gonzalez-Touceda, S. Tovar, C. Diéguez, M.J. Santander-Ortega, M.J. Alonso, Rational design of polyarginine nanocapsules intended to help peptides overcoming intestinal barriers, J. Control. Release. 263 (2016) 4–17. doi:10.1016/j.jconrel.2017.02.024.

[2] S. Klein, The use of biorelevant dissolution media to forecast the in vivo performance of a drug, AAPS J. 12 (2010) 397–406. doi:10.1208/s12248-010-9203-3.

[3] S.B. Bravo, M.E.R. Garcia-Rendueles, S. Perez-Romero, J. Cameselle Teijeiro, J.S. Rodrigues, F. Barreiro, C. V. Alvarez, Expression of exogenous proteins and short hairpin RNAs in human primary thyrocytes, Anal. Biochem. 400 (2010) 219–228. doi:10.1016/j.ab.2010.01.034.
